# Using data science to identify climate change and health adverse impacts and solutions in Africa: a scoping review

**DOI:** 10.1038/s44401-025-00057-w

**Published:** 2026-02-16

**Authors:** Caradee Y. Wright, Anelisa Jaca, Thandi Kapwata, Natasha Naidoo, Babatunde Awokola, Engineer Bainomugisha, Kiros Berhane, Suzana Blesic, Anderson Gwanyebit Kehbila, Rajen N. Naidoo, Bono Nemukula, Benjamin Kofi Nyarko, Akinkunmi Paul Okekunle, Tolu Oni, Reginald Qunasah, Sokhna Thiam, Ibrahim Sidi Zakari, Negussie Beyene, Emile R. Chimusa, Candice Webster

**Affiliations:** 1 Environment and Health Research Unit, South African Medical Research Council, Pretoria, South Africa; 2 Department of Geography, Geoinformatics and Meteorology, University of Pretoria, Pretoria, South Africa; 3 Cochrane South Africa, South African Medical Research Council, Cape Town, South Africa; 4 Environment and Health Research Unit, South African Medical Research Council, Johannesburg, South Africa; 5 Department of Environmental Health, University of Johannesburg, Johannesburg, South Africa; 6 Medical Research Council Unit the Gambia at London School of Hygiene and Tropical Medicine, London, The Gambia; 7 Department of Computer Science, Makerere University, Kampala, Uganda; 8 Department of Biostatistics, Mailman School of Public Health, Columbia University, New York, NY USA; 9 Faculty of Physics, University of Belgrade, Belgrade, Serbia; 10 Stockholm Environment Institute, Nairobi, Kenya; 11 Discipline of Occupational and Environmental Health, University of KwaZulu-Natal, Durban, South Africa; 12 Environmental Health Directorate, National Department of Health, Pretoria, South Africa; 13 University of Cape Coast, Cape Coast, Ghana; 14 Research Institute of Human Ecology, Seoul National University, Seoul, Republic of Korea; 15 Seoul National University, Seoul, Republic of Korea; 16 MRC Epidemiology Unit, University of Cambridge, Cambridge, UK; 17 School of Public Health, University of Ghana, Accra, Ghana; 18 African Population and Health Research Center, West Africa Regional Office, Dakar, Senegal; 19 Abdou Moumouni University, Niamey, Niger; 20 AHRI-APOPO Project, Armauer Hansen Research Institute, Addis Ababa, Ethiopia; 21 Department of Applied Sciences, Faculty of Health and Life Sciences, Northumbria University, Newcastle, Tyne and Wear UK; 22 Environment and Health Research Unit, South African Medical Research Council, Cape Town, South Africa

**Keywords:** Climate change, Diseases

## Abstract

Africa is experiencing the impacts of climate change. While global epidemiological studies using traditional analytical methods to study the relations between climate change and health exist, studies using data science to tackle these topics are increasing. The aim of this study was to identify how data science is being used to understand climate change impacts on health in Africa. We carried out a scoping review to synthesize the evidence of data science applied to understand health outcomes associated with climate change in Africa. Among 100 included articles, several temporal and spatial analytical tools and models were applied to determine the relationships between climate change factors and health outcomes for morbidity and mortality. For example, early warning systems for malaria were the most studied adaptation intervention. Africa has a wealth of evidence for addressing the health impacts of climate change to inform solutions for Africa and other countries around the world.

## Introduction

Climate change has significantly influenced the landscape of human health risks and outcomes in the 21st century^1–3^. Health threats stemming from the impacts of climate change on the environment include meteorological changes that result in an increased frequency and magnitude of extreme weather events, such as floods, droughts, heatwaves, and wildfires^4^. These climate changes are associated with changes in the incidence, prevalence, spatial, and temporal distribution of diseases and adverse health outcomes^5^.

Climate change projections for Africa are considered dire against the backdrop of Africa’s existing challenges, such as poverty, inequality, food insecurity and conflict^6^, exacerbating Africa’s low adaptive capacity to climate change pressures and shocks^7^. Ambient temperatures in Africa have been steadily rising by ~0.3°C/decade between 1991 and 2021, and 2021 was among the top five warmest years on record^8^. Mean ambient temperatures for Africa are projected to be between 4 and 6 °C warmer by 2100^9^, and precipitation is anticipated to change in distribution and intensity across the continent^10^.

Data science may be helpful for Africa to help coordinate and advance African health data infrastructure and initiatives^11^. Improvements in data science may advance research on climate change-related health risks, decision-making, and the prioritization of investment in adaptation interventions for the benefit of public health. In addition, there is a need to evaluate the necessity of climate health adaptation to inform the development of prioritization frameworks for decision-makers. Typically, epidemiological studies have been conducted to understand the associations between climate change and associated health outcomes and impacts on health systems to inform prevention activities and solutions aimed at protecting human health and well-being. Data science plays a crucial role in analyzing complex datasets and generating predictive models that can enhance the accuracy of early warning systems^11^.

Environmental health epidemiology is underpinned by established biostatistical modeling techniques that remain important^12^. However, as data sets grow in diversity, size and complexity, the need for data science techniques, such as machine learning, advanced statistics, and astute data management has arisen^12^. Statistical programming, sophisticated computational modeling and machine learning approaches, working with complex, imperfect, real-world structured and unstructured datasets, and rigorously critiquing data science-based research in environmental health are now critical skills needed when conducting environmental health epidemiological studies^13^.

Recently, data science and computational science have been used in public health sciences^14^. Data science has helped to resolve some of the methodological challenges in environmental health research, for example, high-dimensional outcomes and exposure, and the creation of prediction models (essential when trying to understand climate change). This scoping review synthesizes the existing evidence on how data science has been used to assess the relationships between climate change and health impacts in Africa to provide evidence to inform design and development of appropriate interventions to prevent adverse climate change-related adverse health impacts in Africa. While previous scoping reviews have considered climate change impacts on mental health^15^, child health^16^ and neurological health^17^, none of these reviews have used data science as an inclusion criterion hence our consideration that this may be the first review of its kind.

## Results

### Data science approaches applied in climate change and health research in Africa

The full article extractions are provided in the Supplementary material (Table S3). Among the 100 retrieved and included studies, the data science approaches applied included temporal data analysis (*n* = 28), spatial data analysis (*n* = 10), modeling of temporal data (*n* = 88), modeling of spatial data (*n* = 11) and validation of temporal data (*n* = 4) as illustrated in Tables 1 and 2.

**Table 1:** Summary of data science approaches used in studies that analyzed, modeled or validated temporal data

Type	Method	Health Outcomes
**Analysis of Temporal Data**		
Regression Models	Multivariate regression analysis	Malaria
Standard approaches to data analysis	Mann Kendall trend test	Malaria
	Spearman's correlation test	Malaria, COVID-19, schistosomiasis
	Standard correlations analysis	Malaria, cholera, One Health
	Students *t*-test/Pearsons’s chi square test Poisson distribution	Pulmonary embolism, malnutrition, cholera, malaria, diarrhea
Nonlinear Approaches	ARMA, ANOVA	Measles, cutaneous leishmaniasis
	Univariate Analysis	Atopic dermatitis
PCA	Principal Component Analysis (PCA)	Malaria
Decision Trees	Decision, Classification and Regression Tree Analysis	Malaria, malnutrition, COVID-19
Other	Das Gupta Method	Mortality
	Change-point Analysis	Malaria
	Change and Health Evaluation and Response System (CHEERS)	Morbidity
	Granger Causality Test	Malaria
**Modeled Temporal Data**		
GAM	Generalized Additive Modeling (GAM), Generalized Additive Mixed Models (GAMM), Generalized Additive Model by Livelihood Based Boosting (GAMBOOST)	Meningitis, cholera, malaria, mortality, Rift Valley fever, influenza, COVID-19, Dengue fever
Regression Models, Various	Simultaneous Regression Model	Stunting
	Mixed-effects Poisson Regression Model, Multiple and Multivariate Regressions	Mortality, malaria, malnutrition, measles, cardiovascular and respiratory disease, atopic dermatitis, hospital admissions, birth weight; cardiovascular deaths
	Linear Regression	Stroke, respiratory syncytial virus bronchiolitis (RSV), meningitis, cardiovascular deaths, stunting, malaria, malnutrition
	Linear or Non-Linear Regression	Non-communicable diseases, mortality, heat effects, malaria, years of life lost
	(Poisson) Regression Models	Cholera, cardiovascular deaths, malaria, mortality, diarrhea,
	Logistic Regression Models	Heat effects, mortality
	Negative Binomial Regression Model	Diarrhea
GEE	Generalized Estimating Equations (GEE)	Mortality
GLM	Generalized Linear Models and Mixed Models	Cholera, tuberculosis, meningitis, malaria, vector
Bayesian	Bayesian Modeling	Malaria, West African Rift Valley fever
Nonlinear modeling	Distributed Lag Non-Linear Model	Pneumonia, mortality
	Autoregressive Integrated Moving Average (AIMA)	Influenza, malaria
ENM	Environmental Niche Modeling (ENM)	Malaria, vector
	MaxEnt	Malaria, vector
Other	Ensemble Modeling for Health Data	Meningitis
	MARA Seasonality Model (MSM) for Climate Data	Malaria
	Dynamic Risk Model Based	Rift Valley fever
	Fixed Effects Models (FEM) and Random Effects Models (REM)	Malaria
	RayMan Model	Heat effects, measles
	Vector Autoregressive Moving Average Processes Model (VARMAX)	Malaria
	Framework Method	Diarrhea
	The Space-Time Permutation Model	Diarrhea
	Change and Health Evaluation and Response System (CHEERS)	Morbidity
	Markov Chain Monte Carlo stimulation; Incidence rate ratios (IRR) estimation	Malaria
	Fuzzy Logic Suitability (FLS) Model	Malaria
Author's own	Authors' own methods	Malaria, yellow fever
**Validation of Temporal Data**		
	Deviance Information Criterion (DIC)	Malaria
	Likelihood Ratio Tests	Cardiovascular deaths
	Augmented Dickey–Fuller (ADF) and Kwiatkowski, Phillips, Schmidt and Shin (KPSS) stationarity tests for variables; Autoregressive Distributed Lag (ARDL)–Bounds Test Model for testing causality in non-stationary series	Malaria
	VECM (vector error correction model)	Influenza

**Table 2:** Summary of data science approaches used in studies that analyzed or modeled spatial data

Type	Method	Health Outcome
**Analysis of Spatial Data**		
GIS-based	Geo-referencing using Geographical Information System (GIS)	Diarrhea
	Risk modeling	Cutaneous leishmaniasis
	ArcGIS Predictive Analysis	Vectors
IDW	Inverse Distance Weighted (IDW) Method	Malaria
Other	Kulldorf scanning method with a Monte Carlo algorithm (Multivariate)	Malaria
	SatScan method	Malaria
	FCLIM-Multivariate linear Regression; correlations; spatial interpolations	Malnutrition
	Susceptible-Exposed-Infected-Recovered (SEIR)	Cholera
Correlations	Spatial correlations in a CAR process	Malaria
**Modeling of Spatial Data**		
GLMM	Spatial Generalized Linear Mixed Model (GLMM)	Malaria
Moran's I	Moran's I	Malaria
Bayesian	Bayesian Modeling/Regressions	Malaria, stunting, diarrhea
	Combined Geographical Information System (GIS) and Bayesian belief networks (BBN) - GIS-BBN models	Malaria
Spatial regression	Multiple Regression Analysis	Malaria
Other	Space-time Risk Mapping	Malaria
	Satellite Inversion Models	Macrosomia

### Studies that used both data science and traditional approaches

Of the 100 studies, only one study compared the validity and integrity of the data science methods employed in the analysis. Sehlabana et al.^18^ (Table S3) compared classical and Bayesian methods of estimation when modeling malaria incidence in Limpopo province of South Africa^18^. Classical models used were the Poisson Regression Model, Negative Binomial Model and the Maximum Likelihood Estimation. The Bayesian approach was adopted with the Computation of Negative Binomial Using Bayesian Estimation. Both the Bayesian and the classical frameworks had similar results in terms of a negative relationship between malaria incidence and rainfall. However, the frameworks differed as the Bayesian framework indicates that an increase in Normalized Difference Vegetation Index (NDVI) and day temperature are associated with malaria incidence, whereas the classical framework does not provide evidence of a relationship between these elements. Additionally, the Bayesian framework identified an upward trend in malaria incidence over the study period, while the classical method failed to identify any particular trends.

### Climate change and health impacts findings

**Fig. 1:**
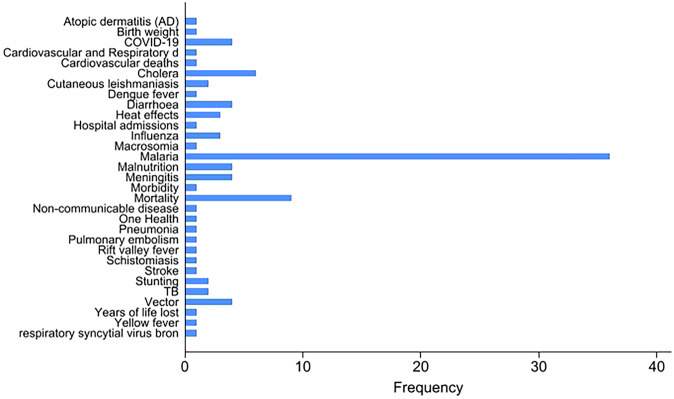
The percentage of diseases studied in the articles.

The greatest number of articles were about malaria followed by all-cause mortality (Fig. 1). Specific findings of associations between climate variables and health outcomes are given in Tables 3–5 and mentioned by communicable and NCDs below. The most commonly used data science methods to study malaria, cholera, diarrhea, and meningitis include generalized additive models (GAM), Poisson and negative binomial regression, generalized linear and mixed models, Bayesian spatial and spatiotemporal models, time series analysis, and spatial analysis tools such as Moran’s I, Maxent, and GIS-based approaches.

**Table 3:** Summary of data science approaches used in the study of health outcomes general morbidity and mortality health outcomes

Health indicator	Climate/Exposure Variable	Data Science Approaches
General Morbidity	Temperature, rainfall, wind, humidity, Global One Health Intrinsic Drivers index that factor in environmental variables, Temperature and rainfall	CHEERS, Standard Approaches to Data Analysis
Heat illness	Heat: Indoor Apparent temperature	Regression Model
	Heat	Regression Model
	Heat	Rayman Model
Years of life lost (YLL)	Daily maximum temperature (High and low)	A distributed lag nonlinear model
Mortality risk	Heat and NO_2_ concentration	Poisson Generalized Estimating Equations (GEE) model
Neonatal mortality	Admission temperature (Cold/heat)	Logistic regression
Child mortality	Heat	Das Gupta Method
Mortality	Temperature, Heat and rainfall	Poisson regression model Generalized Additive Model Lag non-linear model Generalized Additive Model Time series Poisson regression model Distributed Lag Model Multivariate meta-regression model

For a definition of each data science approach, please see the glossary in the Supplementary material A.

**Table 4:** Summary of data science approaches used in the study of non-communicable diseases

Health Outcome	Climate/Exposure Variable	Data Science Approaches
Stunting	Extreme weather events	Regression and Bayesian Models
	Temperature and rainfall	Regression Models
Birth weight	Temperature and rainfall	Regression Models
YLL from NCDs	Heat	Regression Models
Atopic dermatitis (AD)	Temperature, UV index, Rainfall Humidity, Cloud cover	Regression Models; Nonlinear Approaches
Malnutrition	Temperature and Rainfall	Regression Model
	Temperature and Rainfall	Spatial modeling
	Temperature and Rainfall	Statistical modeling
	Temperature and Rainfall	Regression model
Pulmonary embolism	Temperature, Rainfall and Air Pollution	Traditional statistics: multiple binary logistic regression
Macrosomia (Increased Birth Weight)	Household PM	Satellite Inversion Models
Cardiovascular Death	Temperature and Rainfall	Regression Model Likelihood Ratio Poisson Model
Stroke	Heat	Regression Models
Cardiovascular and Respiratory Disease	Heat	Regression Models

For a definition of each data science approach, please see the glossary in the Supplementary material A.

**Table 5:** Summary of data science approaches used in the study of communicable diseases

Health Outcome	Climate/Exposure Variables	Data Science Approach
Malaria	Temperature and rainfall Rainfall and temperature	ENM; MORAN'S 1; GLMM; Seasonality Model (MSM); PCA; Decision Tree; Change Point Analysis; SatScan; ARDL; ADF; KPSS; IDW; Granger Causality; Space Time Risk Mapping; Spatial Correlation; IRR; DIC; Regression Model; Agent-Based; GAM; Bayesian; GLM; Nonlinear Modeling; FLS; Standard Approaches to Data Analysis; Other- FARMAX; FEM; REM; Authors Own; Kulldorf; MARA
Zika virus	Rainfall and temperature and elevation	Spatial Modeling ArcGIS
Dengue fever	Temperature, wind speed, relative humidity	GAM
Schistosomiasis	Diurnal temperatures	Traditional statistics
Vector species	Rainfall and temperature, NDVI, relative humidity	Mixed Bayesian statistical model
Cutaneous leishmaniasis	geographical surface, slope, elevation and annual rainfall	Spatial Risk Analysis
Rift Valley fever	Rainfall, Vegetation Index, Relative Humidity at Maximum And Minimum Temperatures and SST	Other dynamic risk model: GAM Mixed Bayesian statistical model
Yellow fever	Seasonal air temperature, vegetation index, rainfall; temperature suitability index, temperature suitability and rainfall	Authors Own: multilevel logistic regression model to spatio-temporal variability in transmission
Respiratory Syncytial Virus Bronchiolitis (RSV)	Temperature, relative humidity and rainfall	Regression Models
Meningitis	Temperature, rainfall dust status, sunshine hours, max and min temperature, relative humidity, rainfall quantity, wind speed	Regression Model Ensemble Modeling for Health Data GAM
Diarrhea	Temperature, Rainfall, Relative Humidity, Wind Speed and Evaporation	Regression Model; Bayesian; Nonlinear Approaches; Standard Approaches; Other-Framework Method
Tuberculosis	Temperature, Rainfall	GIS Based; GLM
Influenza	Temperature, Rainfall, Relative Humidity, Wind Speed	Statistical modeling: Generalized Additive Model (GAM) and Nonlinear Modeling Time series: Vector Error Correction Model (VECM)
Measles	Air Temperature, Relative Humidity, Wind Speed, Solar Radiation	Regression Models; Rayman; Nonlinear Approaches
Pneumonia	Temperature, relative humidity	Nonlinear Modeling
COVID-19	Temperature, Rainfall, Relative Humidity, Insolation, Topographic Factors	Decision Tree, Standard Approaches to Data Analysis; Gam,
Cholera	Temperature, Rainfall, Relative Humidity, Drought	GAM; Regression Models; GLM; Standard Approaches to Data Analysis; Other- (SEIR)

For a definition of each data science approach, please see the glossary in the Supplementary material A.

### Communicable diseases: vector-borne diseases

Malaria emerged as the most frequently studied vector-borne disease, with 38 articles. Most of these studies found that malaria incidence or prevalence increased during warmer and wetter conditions. Other vector-borne diseases, such as Rift Valley fever^19^, yellow fever^20^, and dengue fever^21^ were each the subject of a single article (Table S3)^19–21^.

### Communicable diseases: diarrhea and cholera

Diarrhea and cholera were reported by four (Seidu et al., Asare et al., Alemayehu et al., Lee et al.,)^22–25^ and six (Mendelsohn et al., Fernández et al., Paz, Jutla et al., Arabi, Charnley)^26–31^ articles respectively. Most cholera studies found that elevated temperatures followed by heavy rain increased the risk of cholera outbreaks (Table S3).

### Communicable Diseases: COVID-19

COVID-19 was reported as a health outcome in four articles (Endeshaw et al., Khalis, et al., Phiri et al., Zio et al., (Table S3)^32–35^. Some of these studies identified increased wind speed, humidity, rainfall, and concentrations of particulate matter and ozone as risk factors for COVID-19 infection.

### Communicable diseases: respiratory diseases (tuberculosis, influenza, pneumonia)

The burden of respiratory diseases such as tuberculosis (TB), influenza, and pneumonia is influenced by climate change. In general, TB incidence increases with rising rainfall and temperature^36^ while influenza tends to spread with lower temperatures and higher relative humidity^37^ (Table S3). Pneumonia prevalence appears as a seasonal phenomenon but historical data is inconsistent and recent studies have shown a marked association between increased hospital admissions and lower temperatures and relative humidity (Table S3)^38^.

### Non-communicable diseases (NCDs)

Cardiovascular conditions such as stroke were reported in three studies that found a strong association between these diseases and increased temperatures, as well as heatwaves (Table S3)^39–41^. Additionally, six articles referenced malnutrition, reduced weight and stunting, conditions that have been linked to high temperatures and below average rainfall (Table S3)^42–47^. Additionally, increased temperature and rainfall, particularly during the first and third trimesters of pregnancy, are positively associated with stunting (Table S3)^43^. One article (Ibekwe et al.) discussed skin diseases, such as atopic dermatitis, which is exacerbated by higher precipitation, humidity, cloud cover, temperature, and ultraviolet (UV) index (Table S3)^48^.

### Climate change and health solutions in data science studies

Of the articles included in the analysis, the majority failed to identify any solutions or interventions. The most common solution (*n* = 10/100 articles) identified was for the data provided to be used to create or improve early warning systems (Alemayehu et al., Luque Fernández et al., Jutla et al., Attaway et al., Batiano et al., Tompkins et al., Kitawa and Asfaw, Faye et al., Ermert et al.)^25,27,29,48–54^. This solution was based on the seasonality of diseases (such as cholera and malaria), and the ability of the articles to model the occurrence of outbreaks. In six articles, (Chapman et al., Jankowska et al., Bunker et al., Diboulo et al., Kulkarni et al., Matthew, 2020)^45,55–59^ (Table S3), while they did not provide a solution or intervention, the authors indicated that the data and outcomes reported in their studies were able to identify risk areas based on climatic and non-climatic factors; hence, this evidence would be instrumental in creating more effective prevention programmes. Finally, two (Lee et al., Wu et al.)^24,60^ (Table S3) articles suggested that campaigns centered on raising awareness or educating the public should be used to improve the understanding of the impacts of weather and climate on health.

### Regional distribution of research activity

**Fig. 2:**
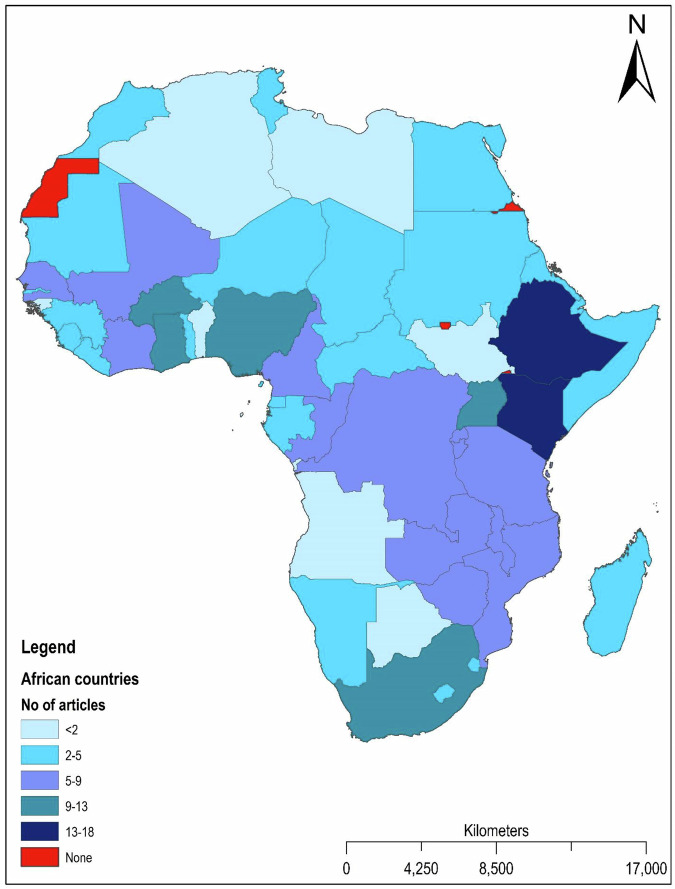
The number of articles in Africa included in this review.

Fig. 2 categorizes the number of articles conducted in each country in the continent, ranging from fewer than two articles to 18 articles. Most climate change-related health articles were from East Africa (28%), particularly in Ethiopia and Kenya, followed by West Africa (28%) and southern Africa (21%).

### First authors’ country of institutional affiliation

**Fig. 3:**
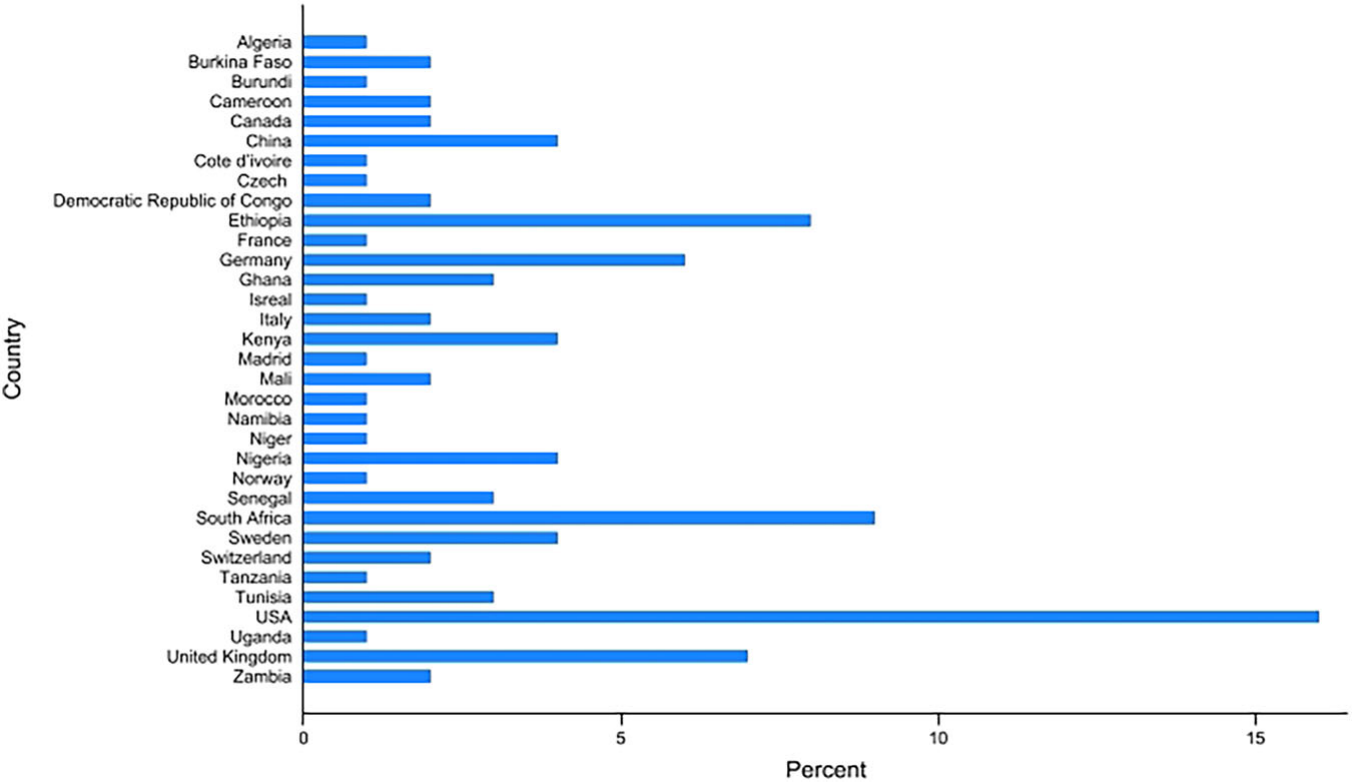
The number of countries in which the first authors had their primary affiliation.

The first authors’ country of institutional affiliation for each article were included in the extracted findings (see Table S3) and indicated that there were more articles published by authors affiliated with institutions based in the United States of America (USA) (16%) than any other country (Fig. 3). Following this was South Africa and Ethiopia that had 9% and 8% of first author affiliations, respectively. Most notably, almost half of all studies (48%) were conducted by authors whose primary affiliation was outside of Africa, predominantly in Europe or the USA.

### Funding supporting data science projects on climate change and health

The majority of the articles presented projects that were funded by donors, organizations and institutions from countries in the northern hemisphere (see the last column of Table S3). Funding originating from the USA and countries from Europe, the European Commission and the UK far exceeded funding originating from African institutions and organizations, e.g., African Academy of Sciences, African Public Health Research Centre and the South African Medical Research Council.

## Discussion

Data science has the capacity to identify and characterize solutions and interventions that can more effectively improve environmental and public health outcomes in Africa^61^. This review explored the data science methods and the health outcomes in response to different climate change exposures. The review is also the first attempt to identify solutions and interventions derived from data science methods by addressing climate change threats to reduce detrimental health outcomes in Africa.

Here, the review of 100 studies applying data science methods to climate change and health research in Africa revealed a strong concentration on communicable diseases, particularly malaria, which was the most frequently studied vector-borne disease. Warmer and wetter conditions were consistently linked to increased malaria incidence, while diarrheal diseases, cholera, and respiratory illnesses such as tuberculosis, influenza, and pneumonia also demonstrated sensitivity to climatic variability. Non-communicable diseases, including cardiovascular conditions, malnutrition, and dermatological disorders, were likewise associated with elevated temperatures, rainfall extremes, and heatwaves. The majority of studies employed advanced modeling techniques—such as Bayesian spatiotemporal approaches, generalized additive models, and GIS-based spatial analyses—underscoring the utility of data science in identifying and quantifying health risks linked to climate variability.

Despite the methodological advances, relatively few studies translated findings into actionable solutions. Among those that did, the most common proposals included strengthening or developing early warning systems for climate-sensitive diseases and leveraging data outputs to identify geographic and population-level hotspots for targeted prevention. However, only a minority of articles addressed public health interventions directly, with some calling for improved awareness campaigns. The analysis highlighted an imbalance in research production: while East and West Africa hosted the largest share of studies, nearly half of the first authors were based outside Africa, primarily in the USA and Europe. This trend was mirrored in funding patterns, where support was predominantly sourced from Northern Hemisphere donors and institutions, reflecting both the global significance of the issue and persistent gaps in African-led capacity and ownership of climate–health research.

Climate change and environmental factors are complex and interactive. The climatic variables and their interactions with health outcomes and disease agents discussed in many of the articles in this study highlighted the complexity of datasets that can be managed by data science methods. Climate change frequently causes simultaneous exposure to several environmental risks, both directly and indirectly, and include variables such as severe temperatures, air pollution, and water scarcity^62^. Data science provides the tools needed to study interactions between complex datasets and understand how they affect health outcomes^63^. Researchers can obtain insights into how multiple environmental factors influence illness risk by constructing models that account for these complex relationships, resulting in evidence to inform more comprehensive and effective health interventions^13^. The scalability of computational data is critical for managing the massive amounts of data required for thorough climate and health assessments^64^. As the volume and complexity of data increase, it is critical to provide scalable computational frameworks capable of handling massive datasets and sophisticated models. Furthermore, resolving dimensionality difficulties, such as choosing essential variables for specific models, is critical for improving the accuracy and usefulness of data science research studies^64^.

Given that climate change is affecting the patterns of communicable diseases and NCDs^65,66^, spatio-temporal analyses of the associations between climate variables and health outcomes is crucial, as it allows us to observe changes in geographical and temporal distribution patterns. This evidence is likely to support climate actions that address the prevalence and geographical extent of diseases and their climatic drivers. Spatio-temporal analysis presents a valuable opportunity to enhance our understanding of how climate change affects disease patterns. By examining the geographical and temporal distribution of climate variables and health outcomes, data scientists can identify trends and correlations that inform targeted climate actions.

Robust data collection and management systems are crucial for applying data science methods to climate change and health. Although there is a growing trend of investment in digital climate health systems, challenges persist in areas such as data quality, completeness, and timeliness^67^. Despite these challenges, opportunities exist to leverage the advancements in digital transformation, including increased connectivity and mobile phone usage. There is also an opportunity to harness data science methods to create and understand new forms of datasets, such as machine learning of seasonal topographical data^68^. The emergence of generative Artificial Intelligence (AI) (large language models) can facilitate interactive collection of a variety of datasets and dissemination of data science solutions to the population in novel ways that are impossible with the current approaches^67,69^.

One of the barriers to effective data science in Africa is the lack of capacity for adequate data infrastructure^70^. Thus, many countries in Africa rely on traditional statistical methods that are less data-intensive, which often results in gaps in the analyzed health data. For instance, access to quality data from hospital records and civil registries is limited, affecting the ability to make evidence-based policy decisions^71^. The reliance on less sophisticated data collection methods exacerbates these issues, making it challenging to develop robust data repositories and predictive models.

The reliance on traditional statistical methods, which are less data-intensive, highlights the lack of access to comprehensive health data (e.g., hospital records, civil registries). Data science techniques require large-scale data. Therefore, investment to support infrastructure and skilled staffing are key elements of developing and maintaining quality data repositories, such as electronic hospital records^61^. Open data initiatives, cross-border and community-driven data collection modalities should also be considered in this endeavor^72^.

Vector-borne diseases are more widely studied in connection with climate change, as temperature, rainfall patterns and humidity can directly influence the habitats and behaviors of vectors leading to shifts in disease transmission seasons^72–74^. Some diseases have seasonal cycles that respond to climatic factors and are well-documented in Africa^75^. However, several non-climatic factors influence transmission as well. Tuberculosis has been recognized as a bacterial disease that is climate sensitive but complicated by several other factors associated with socioeconomic conditions, such as crowded housing and malnutrition, HIV coinfection, diabetes, smoking, alcohol use, and sensitivity of children and the elderly^76^. Thus, the relationship between climate change and diseases that are not transmitted by vectors can be more complex and indirect. Data science is able to use a variety of variables and complex datasets to find links between climatic variables and the spread of diseases by (1) identifying patterns of diseases occurrence in relation to climate, (2) the development of predictive models to forecast future climate conditions and associated disease risk and (3) establishing associations between pathways/mechanisms of disease and climate variables^77^.

While there is a well-established focus on febrile and vector-borne diseases in Africa, particularly in malaria-endemic regions, NCDs have not received the same level of research attention^78^. This imbalance persists even though NCDs are contributing to the disease burden on the continent. The potential of data science to illuminate the impacts of climate change on NCDs, and to identify novel approaches for prevention and management, remains untapped. The viability of data science in identifying the potential impact of climate change on NCDs cannot be underestimated, considering the significance of this phenomenon in exploring unique approaches for addressing the increasing burden of NCDs in Africa^79^. Promoting specialized multidisciplinary research pivots targeted at applying environmental and climate data to harness the gains of data science to address the burden of NCDs in Africa would be promising in addressing this gap. This approach would extend the frontiers of understanding the impact of climate change on NCDs, thereby offering new prevention and treatment opportunities for NCDs management in Africa.

There were some study limitations. The terminology related to climate change and human health, as well as key concepts in this review, is evolving, and new terms may not have been encompassed in our search strategy, which may have resulted in some literature being missing. Within the context of data science, a new and evolving field, there are bound to be new terms associated with these broader topics. While we searched five databases, we may have missed some peer-reviewed literature. In anticipation of this challenge, we searched the reference lists of all included articles to identify any additional literature that may be relevant to the review. Also, we only included English articles and articles written in other languages were not included. Another limitation was that we did not specifically include tropical neglected diseases in our search terms. As this was a scoping review with a very broad topic, we did not conduct a quality assessment of the included articles and this is noted as a limitation. The diversity of data science methods and health outcomes assessed precludes a formal quality assessment or the application of risk of bias tools in this review. We also did not investigate missingness in the data that were presented in the articles included in the review.

The primary focus of this scoping review was on synthesizing existing literature to explore how data science is being applied in public health research, rather than presenting new analytical work by the extensive list of authors. Our contribution lies in the analysis and interpretation of trends across studies, rather than the application of data science techniques ourselves, however, this may be a path for future research.

This scoping review highlights the need for investment into training and retaining data scientists on the African continent to advance a region-specific climate and health agenda. The lack of solutions identified further amplifies the need for more specialized data scientists who will be able to bridge the divide between scientific evidence and action. We also identified the underutilization of data science for automated data analysis which would be a valuable contribution to resource constrained countries.

Finally, this study makes a significant contribution to global public health by synthesizing evidence on how data science methods can illuminate the links between climate variability and health outcomes in Africa, a region disproportionately vulnerable to climate change. By highlighting both communicable and non-communicable disease risks, as well as identifying gaps in intervention design and African-led research, the review provides a foundation for strengthening climate–health surveillance, early warning systems, and adaptation planning. Importantly, the findings underscore the urgency of expanding locally driven, well-funded research and policy responses, ensuring that African institutions are central to generating and applying evidence to protect the health of their populations in the face of accelerating climate risks.

## Methods

**Fig. 4:**
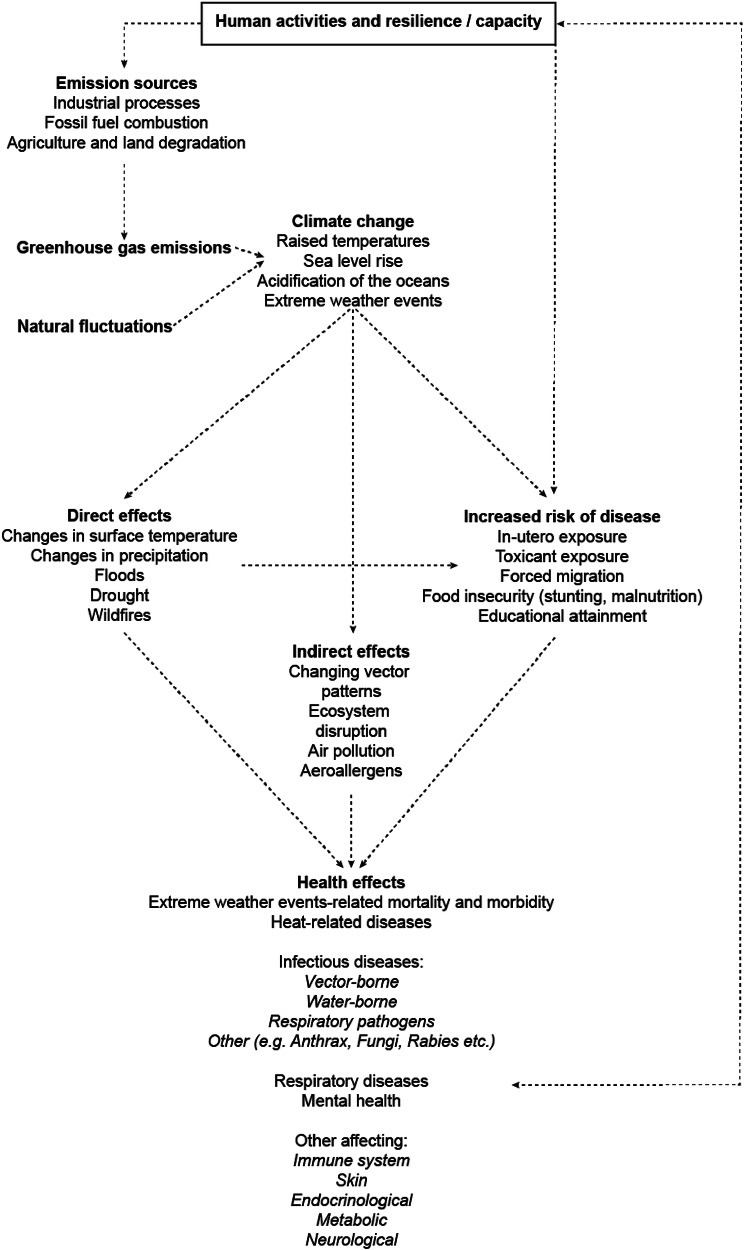
Climate change and health conceptual framework applied in this scoping review.

Our search was informed by the conceptual framework adapted from Helldén et al.^16^ in Fig. 4. Due to the complex nature of the topic and the possible wide range of studies addressing it, a scoping review method was selected using the Joanna Briggs Institute framework^80^ adapted from Arksey and O’Malley^81^ and the Preferred Reporting Items for Systematic Reviews and Meta-Analysis extension for scoping reviews (PRISMA-ScR)^82^, We applied the following steps: (i) developing the objectives and questions; (ii) developing the search strategy; (iii) identifying the inclusion and exclusion criteria; (iv) conducting extraction and charting of results; (v) undertaking expert consultation; (vi) presenting the results; and (vii) developing the discussion, conclusion and implications for research and practice. The protocol was not registered prior to execution of the study.

### Defining data science

We applied the National Institute of Health Strategic Plan for Data Science definition of data science as “the interdisciplinary field of inquiry in which quantitative and analytical approaches, processes, and systems are developed and used to extract knowledge and insights from increasingly large and/or complex sets of data.”^83^ Data science encompasses a wide range of topics, including data, big data, blockchain analytics, digital technology, mobile technology and informatics^63^. It also covers methods useful for spatial data analysis, such as geospatial analysis, image processing and remote sensing^84,85^. Data analytics, artificial intelligence algorithms, complex systems approaches and complex networks algorithms uncover patterns and solve complex problems^86^. Additionally, data science methodologies include time series analysis, computational analysis and modeling and regression analysis and modeling, which include multi-level modeling and time-to-event analysis (please see the glossary in the Supplementary material A file for a definition of each of these terms)^87^.

### Defining climate change

Long-term significant changes in temperature and weather patterns are referred to as climate change^88^. Natural processes, such as fluctuations in the solar cycle, sea spray and volcanic eruptions occur. However, since the 1800s, human activities, primarily the combustion of fossil fuels like coal, oil and gas, have been the primary cause of climate change^89^.

### Defining human health

The World Health Organization defines human health as “a state of complete physical, mental and social well-being and not merely the absence of disease or infirmity”.^90^ Regarding health outcomes, we used the definition of general morbidity to denote a morbidity outcome as “a value describing the presence of disease in the population, or the degree of risk of an event”.^91^ We used the mortality rate definition of “a measure of the frequency of deaths in a defined population over a certain period of time”.^76^ The health outcomes included in this review include non-communicable (NCDs), communicable diseases and all-cause mortality. Non-communicable diseases are commonly referred to as chronic diseases and are non-transmissible diseases of often long duration, including mental health conditions, stroke, heart disease, cancer, diabetes and chronic lung disease^92^. Communicable diseases encompass contagious diseases caused by transmission of an infective agent and include vector-borne diseases such as malaria or Rift Valley fever^93^.

### Eligibility criteria

We followed the PECOS (Population, Exposure, Concept/Context, Outcome and Study design)^94^ framework in identifying our eligibility criteria. The framework guidance was discussed with an information specialist. Studies eligible for inclusion had to meet the following criteria:

- Population: Studies conducted in Africa with an emphasis placed on groups who are vulnerable to the impacts of climate change, e.g., infants and children (infants and children are different, primarily in their age and developmental stage; infants are typically defined as babies from birth up to 1 year old, while children encompasses a broader age range, generally from infancy through puberty), older persons, people with pre-existing/chronic diseases, and outdoor workers.
- Exposure: Studies that assessed environmental factors, agents or exposures related to climate change, such as heat, heatwaves, temperature, floods, and droughts.
- Concept/Context: Literature focused on climate change in an African setting.
- Outcome: Outcomes related to climate change, including climate change-related morbidities or mortalities.
- Study designs: Primary studies/original research (qualitative and quantitative) that used or investigated the use of data science to address health outcomes caused by climate change.

### Exclusion criteria

Articles were excluded for the following reasons:

- Population: Studies conducted in populations outside the African continent.
- Exposure: Studies that failed to assess environmental factors related to climate change, such as pesticides and heavy metals.
- Concept/Context: Studies that excluded countries in Africa or the African continent.
- Outcomes: Studies that did not report human health impacts.
- Study designs/type of article: Studies that did not apply data science. Conference abstracts, books, book chapters, book reviews, protocols, animal studies, and studies without full text.

### Search strategy

We first conducted a pilot search strategy with an information specialist to enhance our search precision. A comprehensive literature search was then conducted after the pilot search in five relevant electronic databases selected to cover both health and climate topics: Web of Science Core Collection (accessed via Web of Science); Scopus (accessed via Elsevier); CAB Abstracts (accessed via Web of Science/OVID); MEDLINE; and EMBASE. The search period was from the inception of the database (no limitations) to 18 October 2023 for articles written in English. In addition, reference lists of eligible peer-reviewed articles were manually searched for additional articles relevant to the scoping review. The databases were searched for relevant articles using text words and Medical Subject Headings (MeSH) terms as part of the search strategy for data science, climate change, and solutions to prevent adverse associated human health outcomes, as defined above, including all literature added to the database until the date of the search (i.e., 31 July 2023). The text words and MeSH terms included those identified below while the full set of search terms is included in the Supplementary material (Table S1):

- Methods: Methods like data science, crowdsourcing, artificial neural networks, database systems, classification techniques, machine learning and more.
- Exposures: Climate change, extreme weather events, natural disasters (i.e., drought, floods, landslides, fires, dust storms or windblown dust, heatwaves, hurricanes, tropical cyclones, storm surges and more).
- Outcomes: Health impacts, such as mortality, morbidity, diet, nutrition, mobility, injury, and mental health.
- Solutions: Interventions, implementation, climate adaptation, adaptive capacity, and resilience.

### Study selection

**Fig. 5:**
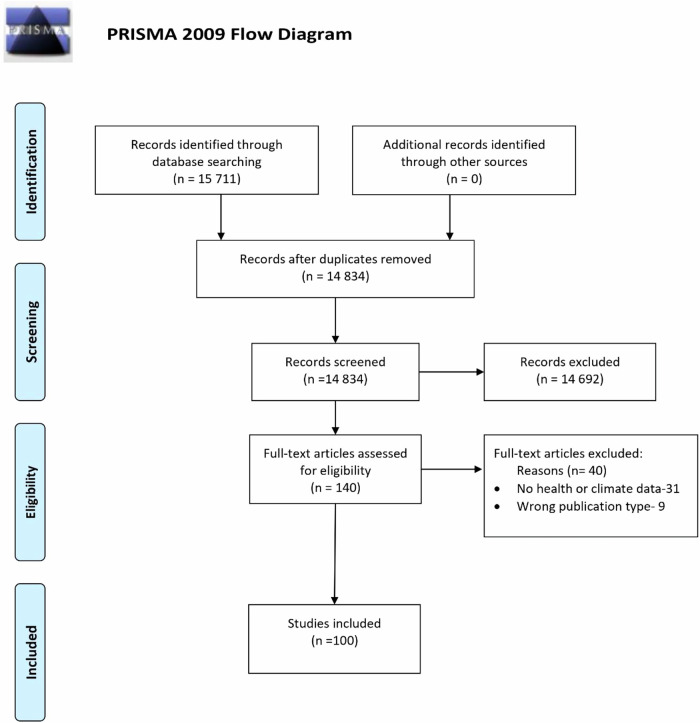
PRISMA diagram illustrates the included and excluded articles in the different phases of the scoping review.

The search results were consolidated, checked and managed in EndNote^TM^ version 20^95^ and Rayyan (https://www.rayyan.ai/)^96^. Rayyan was used for semi-automation of the initial screening of titles and abstracts. Four reviewers (C.W., A.J., S.M., and T.K.) independently screened the titles and abstracts for relevance against the inclusion and exclusion criteria listed above. Subsequently, the authors independently screened the full texts of potentially eligible articles for inclusion and exclusion. During the article selection phase, disagreements between two authors were resolved through discussion with another author (C.Y.W.) to reach a consensus. We initially retrieved 15,711 records. Following deduplication, the remaining 15,249 records were then imported to an online reviewing platform called Rayyan, where a second round of deduplication removed 415 duplicates. The remaining 14,834 records were screened by four reviewers (C.W., T.K., A.J., S.M., C.Y.W.) who independently assessed the title and abstract of each record for inclusion. Full texts were sourced for 140 records that met the inclusion criteria. The full texts were assessed for eligibility and if they failed to meet the eligibility criteria, the reason for exclusion was as detailed in the PRISMA diagram (Fig. 5). This resulted in 100 articles being included in the final review. Since this was a scoping review, articles were not assessed for methodological quality or risk of bias as is done for systematic reviews. This is aligned with our aim to identify and map existing literature on the use of data science to address climate change-related health problems in Africa.

### Data charting and synthesis

All authors assisted to independently extract the article data using a piloted data extraction form in Microsoft Word ^TM^. Data extracted from eligible studies included the surname and initial of the first author, year of publication, the first author’s country of affiliation, the country where the study was conducted, study design, study period, climate change variables used, health outcomes, data science technique (method), suggested intervention or solutions (if any) and key findings (i.e., associations between climate change and human health).

### Analysis

The charted data were checked and categorized by three authors (C.W., S.B., and C.Y.W.) to ensure uniformity in the reporting of outcomes for effective tabulation. Data synthesis involved analyzing findings from the identified articles using thematic analysis approaches. Since the scope of data science is vast, the included studies were broadly categorized. We categorized articles by data science method and then we applied three high-level categories for reporting health outcomes: all-causes, non-communicable diseases (NCDs) and communicable diseases. Where possible, we considered sex-based differences and other socio-demographic characteristics (i.e., socio-economic status, age) mentioned in the studies.

## Supplementary information

Supplementary Information

## Data Availability

All the data reported in this review article are included in the body of the manuscript and in the supplementary tables.
