# Supplementary material for: Using data science to identify climate change and health adverse impacts and solutions in Africa: a scoping review
Source: Npj Health Syst. 2026 Feb 16;3:16. doi: 10.1038/s44401-025-00057-w (PMC13354214; doi:10.1038/s44401-025-00057-w)
Supplement: Supplementary file 1 — Supplementary Information [file 44401_2025_57_MOESM1_ESM.docx]

Table S1. Detailed search terms.

| **Geography** | **Methods** | **Exposures** | **Outcomes** | **Solutions** |
| --- | --- | --- | --- | --- |
| “Africa” OR “Algeria” OR “Angola” OR “Benin” OR “Botswana” OR "Burkina Faso" OR “Burundi” OR “Cameroon” OR "Cape Verde" OR "Central African Republic" OR “Chad” OR "Democratic Republic of Congo" OR "Republic of Congo" OR "Cote d'Ivoire" OR “Djibouti” OR “Egypt” OR "Equatorial Guinea" OR “Eritrea” OR “Ethiopia” OR “Gabon” OR “Gambia” OR “Ghana” OR “Guinea” OR “Guinea Bissau” OR “Kenya” OR “Lesotho” OR “Liberia” OR “Libya” OR “Madagascar” OR “Malawi” OR “Mali” OR “Mauritania: OR “Mauritius” OR “Morocco” OR “Mozambique” OR “Namibia” OR “Niger” OR “Nigeria” OR “Reunion” OR “Rwanda” OR "Sao Tome and Principe" OR “Senegal” OR “Seychelles” OR "Sierra Leone" OR “Somalia” OR "South Africa" OR "South Sudan" OR “Sudan” OR “Swaziland” OR “Tanzania” OR “Togo” OR “Tunisia” OR “Uganda” OR “Zambia” OR “Zimbabwe” OR “Southern Africa” OR “East Africa” OR “Central Africa” OR “Northern Africa” OR “West Africa” | “Data Science” OR "Artificial intelligence" [MeSH] OR “AI” OR " Machine learning" OR “Machine Learning” [MESH] OR "Machine intelligence" OR "Deep learning" OR "Deep learning" [MeSH] OR “Modelling” OR “Satellite imag*” OR “remote sens*” OR “Computer aided vision” OR “Big data” | “Global warming" OR "global warming" [MeSH] OR "Global heating" OR “Heat” OR “Heatwaves” OR “Temperature” OR "climate change" [MeSH] | “Health” OR “Impact” OR “Mortal*” OR “Death” OR “morbid*” OR “Food-related” OR “Agric* OR “Diet” OR “Mobility-related” OR “Injury” OR “Respiratory” OR “Cardiovascular” OR  “psychological health” OR “mental health” | “Intervention” OR “Adaptation” OR “Climate adaptation”: OR “Implementation” OR “Action” OR “Modification” OR “Alteration” OR “Solution” |

**Table S2. Preferred Reporting Items for Systematic reviews and Meta-Analyses extension for Scoping Reviews (PRISMA-ScR) Checklist**

| **SECTION** | **ITEM** | **PRISMA-ScR CHECKLIST ITEM** | **REPORTED ON PAGE #** |
| --- | --- | --- | --- |
| **TITLE** | | | |
| Title | 1 | Identify the report as a scoping review. | 1 |
| **ABSTRACT** | | | |
| Structured summary | 2 | Provide a structured summary that includes (as applicable): background, objectives, eligibility criteria, sources of evidence, charting methods, results, and conclusions that relate to the review questions and objectives. | 4 |
| **INTRODUCTION** | | | |
| Rationale | 3 | Describe the rationale for the review in the context of what is already known. Explain why the review questions/objectives lend themselves to a scoping review approach. | 7 |
| Objectives | 4 | Provide an explicit statement of the questions and objectives being addressed with reference to their key elements (e.g., population or participants, concepts, and context) or other relevant key elements used to conceptualize the review questions and/or objectives. | 7 |
| **METHODS** | | | |
| Protocol and registration | 5 | Indicate whether a review protocol exists; state if and where it can be accessed (e.g., a Web address); and if available, provide registration information, including the registration number. | 7 |
| Eligibility criteria | 6 | Specify characteristics of the sources of evidence used as eligibility criteria (e.g., years considered, language, and publication status), and provide a rationale. | 9 |
| Information sources* | 7 | Describe all information sources in the search (e.g., databases with dates of coverage and contact with authors to identify additional sources), as well as the date the most recent search was executed. | 10 |
| Search | 8 | Present the full electronic search strategy for at least 1 database, including any limits used, such that it could be repeated. | 10 |
| Selection of sources of evidence† | 9 | State the process for selecting sources of evidence (i.e., screening and eligibility) included in the scoping review. | 11 |
| Data charting process‡ | 10 | Describe the methods of charting data from the included sources of evidence (e.g., calibrated forms or forms that have been tested by the team before their use, and whether data charting was done independently or in duplicate) and any processes for obtaining and confirming data from investigators. | 11 |
| Data items | 11 | List and define all variables for which data were sought and any assumptions and simplifications made. | 11-12 |
| Critical appraisal of individual sources of evidence§ | 12 | If done, provide a rationale for conducting a critical appraisal of included sources of evidence; describe the methods used and how this information was used in any data synthesis (if appropriate). | 21 |
| Synthesis of results | 13 | Describe the methods of handling and summarizing the data that were charted. | 12 |
| **RESULTS** | | | |
| Selection of sources of evidence | 14 | Give numbers of sources of evidence screened, assessed for eligibility, and included in the review, with reasons for exclusions at each stage, ideally using a flow diagram. | 12 |
| Characteristics of sources of evidence | 15 | For each source of evidence, present characteristics for which data were charted and provide the citations. | 12 |
| Critical appraisal within sources of evidence | 16 | If done, present data on critical appraisal of included sources of evidence (see item 12). | - |
| Results of individual sources of evidence | 17 | For each included source of evidence, present the relevant data that were charted that relate to the review questions and objectives. | Supp material |
| Synthesis of results | 18 | Summarize and/or present the charting results as they relate to the review questions and objectives. | 12-13 |
| **DISCUSSION** | | | |
| Summary of evidence | 19 | Summarize the main results (including an overview of concepts, themes, and types of evidence available), link to the review questions and objectives, and consider the relevance to key groups. | 16-17 |
| Limitations | 20 | Discuss the limitations of the scoping review process. | 20 |
| Conclusions | 21 | Provide a general interpretation of the results with respect to the review questions and objectives, as well as potential implications and/or next steps. | 21 |
| **FUNDING** | | | |
| Funding | 22 | Describe sources of funding for the included sources of evidence, as well as sources of funding for the scoping review. Describe the role of the funders of the scoping review. | 22 |

JBI = Joanna Briggs Institute; PRISMA-ScR = Preferred Reporting Items for Systematic reviews and Meta-Analyses extension for Scoping Reviews.

* Where *sources of evidence* (see second footnote) are compiled from, such as bibliographic databases, social media platforms, and Web sites.

† A more inclusive/heterogeneous term used to account for the different types of evidence or data sources (e.g., quantitative and/or qualitative research, expert opinion, and policy documents) that may be eligible in a scoping review as opposed to only studies. This is not to be confused with *information sources* (see first footnote).

‡ The frameworks by Arksey and O’Malley (6) and Levac and colleagues (7) and the JBI guidance (4, 5) refer to the process of data extraction in a scoping review as data charting*.*

§ The process of systematically examining research evidence to assess its validity, results, and relevance before using it to inform a decision. This term is used for items 12 and 19 instead of "risk of bias" (which is more applicable to systematic reviews of interventions) to include and acknowledge the various sources of evidence that may be used in a scoping review (e.g., quantitative and/or qualitative research, expert opinion, and policy document).

*From:* Tricco AC, Lillie E, Zarin W, O'Brien KK, Colquhoun H, Levac D, et al. PRISMA Extension for Scoping Reviews (PRISMAScR): Checklist and Explanation. Ann Intern Med. 2018;169:467–473. [doi: 10.7326/M18-0850](http://annals.org/aim/fullarticle/2700389/prisma-extension-scoping-reviews-prisma-scr-checklist-explanation).

**Table S3: Summary of all included articles**

| **First author (Year)** | **Author’s country** | **Country of study** | **Climate change exposure (source, variable where possible)** | **Health outcomes (source, variable)** | **Region** | **Method** | **Key Findings** | **Funder(s) of the study** |
| --- | --- | --- | --- | --- | --- | --- | --- | --- |
| Abdussalam, 2014 | Nigeria | Nigeria | Rainfall, temperature | Meningitis | West Africa | The models employ Poisson generalized additive modeling (GAM) | The results suggest future temperature increases due to climate change have the potential to significantly increase meningitis cases in both the early (2020–35) and late (2060–75) twenty-first century, and for the seasonal onset of meningitis to begin about a month earlier on average by late century, in October rather than November. | The Nigerian Tertiary Education Trust Fund, the National Center for Atmospheric Research, and the National Science Foundation (NSF Award 1211668) provided funding. |
| Abrha, 2019 | Ethiopia | Ethiopia | Diurnal temperature range (DTR), temperature seasonality, precipitation of warmest quarter, precipitation of wettest month, altitude and mean temperature of the warmest quarter | Malaria | East Africa | The maximum entropy model (Maxent) was used to predict malaria transmission risk under climate change. | Malaria transmission increases when precipitation of warmest quarter, temperature seasonality, mean temperature of driest quarter, mean temperature of the warmest quarter, temperature annual range and annual mean temperature increases. | None reported / none to report |
| Adu-Prah, 2015 | Ghana | Ghana | Maximum annual temperature, minimum annual temperature, annual relative humidity, and annual rainfall from 1995 to 2006. | Malaria | West Africa | Spatial generalized linear mixed model (GLMM) and local Moran's I. | The results indicated that temperature and humidity have some association with malaria prevalence in Ghana. Although annual rainfall in the model was found to be less significant, there is evidence of rainfall as a predictor of malaria in Ghana. High annual malaria prevalence coincides with maximum temperature and relatively high rainfall while low prevalence years coincide with low rainfall and low humidity. The intensity and timing of the seasonal peak in each year appears to follow variability in temperature and rainfall. | None reported / none to report |
| Alemayehu, 2020 | Ethiopia | Ethiopia | Monthly total rainfall and monthly average temperature | Diarrhea | East Africa | Mann Kendall trend test and Spearman's correlation test; The space-time permutation model; negative binomial regression model. | The risk of childhood diarrhea increased by 16.66% (RR: 1.1666; 95% CI: 1.164–1.168) per increase in 1 °C temperature; childhood diarrhea increased by 0.16% (RR: 1.00167; 95% CI: 1.001306–1.001928) per 1 mm increase in rainfall; dry season was found to be a high-risk period with excess childhood diarrhea. | None reported / none to report |
| Alene, 2017 | Ethiopia | Ethiopia | Rainfall and temperature | Tuberculosis | East Africa | TB data were geo-referenced and linked using GIS. | The spatio-temporal transmission of childhood TB was found to be associated with district level socio-climatic factors such as urbanization, lower educational status, a high percentage of internal migration, high temperature and high rainfall. | None reported / none to report |
| Amondo, 2023 | German | Uganda | Extreme weather events | Stunting | East Africa | Simultaneous regression model was applied for causal inference accounting for households’ adaptive responses. | Droughts reduced calorie, protein and zinc supply, and overall diet diversity by 67%, 37%, 28% and 30%, respectively. They further traced the effects of this reduced calorie and nutrient availability on child health indicators. A 10% decrease in zinc supply decreased height-for-age z-scores (HAZ) by around 0.139 - 0.164 standard deviations (SD), and increased probability of stunting ranging from 3.1 to 3.5 percentage points. | Projekt DEAL |
| Arab, 2014 | USA | Multiple | Source: National Oceanic and Atmospheric Administration’s (NOAA) National Climate Data Center (NCDC) Variables: mean station pressure, mean sea level pressure, mean temperature, departure of temperature from long term station average, mean vapor pressure, number of days with precipitation at least 1 mm, total precipitation, and departure of precipitation from long term station average. | Malaria | West Africa | Hierarchical, Bayesian statistical spatio-temporal modelling framework. | A statistically significant negative association between malaria rates and average annual temperature and total annual precipitation was found. | None reported / none to report |
| Arabi, 2019 | Cameroon | Cameroon | Variable: Rainfall, temperature, relative humidity | Cholera | Central Africa | Generalized Additive Modeling (GAM) | Increasing cholera incidence was associated with warmer temperature, and increased rainfall. | University of Maroua, DOVE-Project at Johns Hopkins Bloomberg School of Public Health |
| Asare, 2022 | USA | Ghana | Source: -Climate Hazards group Infrared Precipitation with Stations (CHIRPS)  Variables: Monthly mean, minimum, and maximum temperature-2-meter air temperature | Diarrhea | West Africa | Hierarchical Bayesian spatiotemporal modeling framework. | Significant associations between climate variables and diarrhea risk were found. Minimum temperature and diurnal temperature range were mostly positively correlated with diarrhea in the Southern part of the country. Diurnal temperature range had the strongest association with diarrhea with a 1 SD decrease was associated with a 2% increase in diarrhea incidence. However, the strength and direction of associations differed across the four agro-ecological zones. | National Institutes of Health/National Institute of Allergy and Infectious Diseases |
| Attaway, 2017 | USA, Canada | Global | Elevation below 1800m, precipitation level, annual mean temperature between 10 and 30°C | Vector | East, West and sub-Saharan Africa | ArcGIS Predictive Analysis Tools | Aedes mosquitoes are likely to live year-round across many tropical areas in the Americas, Africa, and Asia. | None reported / none to report |
| Bakhtsiyarava, 2018 | USA | Kenya and Mali | Temperature and Rainfall from Integrated Public Use Microdata Series (IPUMS)-Terra | Birth weight | East Africa and West Africa | Multilevel regression models were employed to analyze association between climate variability and birth weight for the most common agricultural specializations: food cropping, cash cropping, and pastoralism. | There are differences in sensitivity to climate among different agricultural communities. An additional 100 millimeters of rainfall during the 12-month period before birth was associated with a 47-gram (P = .001) and 89-gram (P = .10) increase in birth weight for food croppers in Kenya and Mali, respectively. Every additional hot month in food-cropping communities in Kenya was associated with a 71-gram decrease in birth weight (P = .030), likely because of food croppers’ limited use of modern agricultural techniques. Overall, cash croppers are least sensitive to climate variability in both countries. | Minnesota Population Center (R24 HD041023) support funded through grants Eunice Kennedy Shriver National Institute for Child Health and Human Development; National Science Foundation–funded Terra Populus project (NSF award ACI-0940818) |
| Barteit, 2023 | Germany; Burkina Faso; Kenya | Burkina Faso | Indoor temperature, Relative humidity; Outdoor temperature, Precipitation, Wind speed, Wind direction, Solar radiation; Remote sensing-based land use and land cover classifications, surface water | Morbidity | West Africa | Change and Health Evaluation and Response System (CHEERS) as a methodological framework using a multi-tiered approach to assess health and environmental exposures at the individual, household, and community levels, utilizing digital tools such as wearable devices, indoor temperature and humidity measurements, remotely sensed satellite data, and 3D-printed weather stations. | The use of wearables to study the impact of extreme weather on health has shown significant effects of heat exposure on sleep and daily activity, highlighting the urgent need for interventions to mitigate adverse health consequences. | German Research Foundation (Deutsche Forschungsgemeinschaft–funded research unit (Forschungsgruppe FOR 2936) |
| Bationo, 2021 | France | Burkina Faso | Source:  European Center for Medium-Range Weather Forecasts (ECMWF) fifth-generation reanalysis ERA5; variables: rainfall -mean daily average temperature -mean daily minimum -mean daily maximum temperature -mean daily average wind speed mean daily average relative humidity -mean daily average atmospheric pressure; mean daily average cloud cover -mean daily thermal amplitude | Malaria | West Africa | Kulldorf scanning method with a Monte Carlo algorithm (purely spatial analysis) to detect high risk clusters; generalized additive model with a negative binomial distribution and a smoothing spline function; generalized additive mixed model (GAMM). | Malaria incidence was positively correlated with cumulative rainfall, humidity, cloud cover, and number of rainy days, and negatively correlated with thermal amplitude. Malaria incidence was positively correlated with temperature and negatively correlated with atmospheric pressure; Relationship between rainfall and the number of cases was quasi-linear–-Relationship between the number of malaria cases and temperature was non-linear. | French Initiative 5%—Expertise France; Endowment Fund through the NGO Prospective et Coopération; French Embassy in Burkina Fasso |
| Bettaieb, 2020 | Tunisia, France | Tunisia | Daily temperature, relative humidity, wind speed and direction and sea level pressure from the weather station at the Tunis Carthage airport station) Daily air pollution (NO2) from National Environmental Protection Agency from an urban fixed monitoring | Mortality | North Africa | Poisson Generalized Estimating Equations (GEE) model | The estimated breakpoint was 31.5 ˚C (standard deviation: 0.9 ˚C). After adjustment for potential confounders, the daily mortality increased significantly by 2.00% [95% confidence interval: 0.68– 3.16] for a 1 ˚C increase in daily maximum temperature above the breakpoint. An increase of 10 mg/m^3^ in NO2 was associated with a significant increase in daily mortality (0.48% [0.08–0.88]). | 6th EU Framework Program CIRCE: Climate Change and Impact Research: The Mediterranean Environment [Grant number: 036961] |
| Bishop-Williams, 2018 | Canada, Uganda | Uganda | Daily rainfall and maximum, minimum, and average temperature From the European Centre for Medium-Range Weather Forecasts Re-analysis (ERA)-Interim Climate Database | Hospital admissions | East Africa | Mixed-effects Poisson regression model | Admission counts were highest for acute respiratory infections, malaria, and acute gastrointestinal illness, which are climate-sensitive diseases. Hospital admissions were 1.16 (95% CI: 1.04, 1.31; p = 0.008) times higher during extreme high temperatures (i.e., >95th percentile) on the day of admission. Hospital admissions association with season depended on year; admissions were higher in the dry season than the rainy season every year, except for 2014. | International Development Research Centre, Canadian Institutes of Health Research, National Sciences and Engineering Research Council, and the Social Sciences and Humanities Research Council of Canada |
| Bo, 2023 | China | Global | Source: Climate Research Unit Variable: annual average temperature | Stroke | All of Africa | Linear regression, random effects model | Global burden of stroke deaths attributable to high temperature increased between the period 1990 to 2019. Stroke related mortality attributed to high temperatures was higher among males than females. Stroke burden attributable to high temperature was higher in the elderly population. | National Natural Science Foundation of China Natural Science Foundation of China Nanshan District Science and Technology Program Key Project |
| Brini, 2018 | Tunisia | Tunisia | Source: Tunisian Ministry of Agriculture.  Variables: average temperature, average humidity, average rainfall | Respiratory Syncytial Virus bronchiolitis (RSV) | North Africa | Linear regression | Average temperature had the strongest association with RSV bronchiolitis; RSV bronchiolitis decreased with the increasing temperature. No correlation between RSV and rainfall Inverse correlation between humidity and RSV. | Ministry of Higher Education and Scientific Research in Tunisia |
| Bunker, 2017 | Germany, Sweden and Burkina Faso | Burkina Faso | Maximum daily temperature | Non- communicable disease (NCD) | West Africa | Daily time series regression analysis | Moderate 4-day cumulative rise in maximum temperature from 36.4°C (50th percentile) to 41.4°C (90th percentile) resulted in 4.44 (95% CI 0.24 to 12.28) excess daily NCD and Years of Life Lost for all ages, rising to 7.39 (95% CI 0.32 to 24.62) at extreme temperature (42.8°C; 99th percentile). Premature death from non-communicable diseases was elevated significantly with moderate and extreme heat exposure. | Klaus-Tschira Stiftung gGmbH |
| Cavallin, 2020 | Italy | Mozambique | Axillary temperatures from the day of admission | Mortality | Southern Africa | Logistic regression c-index | Being cold at admission and becoming cold or hyperthermic at day 1 were associated with increased likelihood of mortality. | Italian Agency for Development Cooperation (grant AID 11497/CUAMM/MOZ - Healthy Newborn Project: Innovative approaches in protecting the health of the newborn in the Province of Sofala). |
| Chapman, 2022 | United Kingdom | Africa | Anthropogenic emissions using the Detection and Attribution Climate Model Intercomparison (DAMIP) experiment of Coupled Model Intercomparison Project Phase 6 (CMIP6). | Mortality | All | Mathematical models and linear equations to estimate heat-related child (under-5) mortality using Das Gupta Method to decompose the contribution of changes in climate, population, and all-cause mortality rate to changes in heat-related child mortality. | Between 1995 and 2004, the CMIP6 ensemble mean heat-related child mortality in Africa was approximately 7000–11 000 deaths per year, depending on whether a lower (coefficient = 0.61) or higher (coefficient = 1.0) sensitivity to heat was used. Without climate change, this would have been approximately 4000–6000 deaths per year.  Heat-related child mortality in Africa is projected to increase over the next 30 years as temperatures rise. | Natural Environment Research Council (NERC) (Grant Numbers NE/T013613/1, NE/T01363X/1); Research Council of Norway (RCN) (Grant Number 312601). The Swedish Research Council for Health, Working Life and Welfare in collaboration with the Swedish Research Council (Forte) (Grant Number2019-01570); and the National Science Foundation (NSF) (Grant Number ICER-2028598); coordinated through a Belmont Forum partnership. Marsham was also funded by HyCRISTAL, IMPALA and the NCASA CREW programme. |
| Charnley, 2021 | UK | Africa | Drought | Cholera | All | Generalized linear models | Future projections highlighted the potential for sustainable development gains to offset drought-related impacts on cholera risk. | Natural Environmental Research Council; MRC |
| Chen, 2023 | USA | Global | Maximum temperature and precipitation from The Coupled Model Intercomparison Project Phase 6 (CMIP6) data | Meningitis | All | Generalized linear regression models; multi model ensemble. | Among all significant factors, temperature variability demonstrated the most notable effect (1.04, 95 % CI: 1.03–1.04, p < 0.001) on meningitis incidence, compared to that of precipitation (0.99, 95 % CI: 0.99–0.99, p < 0.001) and wind speed (1.02, 95 % CI: 1.02–1.02, p < 0.001). | None reported / none to report |
| Diboulo, 2012 | Switzerland | Burkina Faso | Lags of weather; temperature, cumulative rainfall | Mortality | West Africa | Time series Poisson regression | Mortality patterns in the Nouna HDSS appear to be closely related to weather conditions. | The authors have not received any funding or benefits from industry or elsewhere to conduct this study. |
| Dukić, 2012 | United States of America | Ghana | Source: Ghana Meteorological Services  Variables: dust status, sunshine hours, max and min temperature, relative humidity, rainfall quantity, wind speed | Meningitis | West Africa | Generalized additive models | The higher levels of current month temperature, sunshine, and the percentage of dusty days, as well as previous month’s CO emissions and wind, tend to co-occur with the higher meningitis incidence. | None reported / none to report |
| Egondi, 2012 | Sweden | Kenya | Meteorological data on temperature and rainfall were obtained from the Meteorological Department of Kenya for the period of 2003–2008. | Mortality | East Africa | Poisson regression model  Generalized Additive Model | Increases in mortality were associated with both hot and cold weather as well as rainfall in Nairobi, but the relationship differed with regard to age, sex, and cause of death. | Umea Centre for Global Health Research, with support from FAS, the Swedish Council for Working Life, and Social Research (grant no. 2006-1512). |
| Endeshaw, 2022 | Ethiopia, Australia and USA | Ethiopia | Climatic factors such as temperature, rainfall, relative humidity, sunshine duration, and wind speed | COVID-19 | East Africa | Spearman rank correlation test | Climatic factors such as humidity, rainfall, and wind speed influence the transmission of COVID-19 in Addis Ababa. COVID-19 cases have shown seasonal variations with the highest number of cases reported during the rainy season and the lowest number of cases reported during the dry season. | None reported / none to report |
| Ermert, 2013 | Germany | Africa | Seasonality – temperature and precipitation | Malaria | All | Climate-driven seasonality models | West Africa: Decreased length of the malaria season is projected. Northern Sahel: No longer suitable for malaria in the projections. Various areas farther South: Shorter malaria seasons are expected. East Africa: Higher temperatures and nearly unchanged precipitation patterns lead to longer transmission seasons and an extension of highland malaria territories in the model simulations. | Federal German Ministry of Education and Research (BMBF) in the GLOWA program. Ministry of Innovation, Science, Research and Technology (MIWFT) of the federal state of North Rhine-Westphalia. European Commission’s Seventh Framework Research Program. |
| Faye, 2021 | Senegal | Senegal | Daily weather station data from Kedougou station (17km from Banda Fassi, Senegal) daily minimum, maximum, and mean temperatures (˚C), dew point temperature (˚C), wind speed (m/s), and precipitation (mm) | Mortality | West Africa | Generalized additive model and distributed lag non-linear model to investigate the effect of heat wave on mortality and to evaluate the nonlinear association of heat wave definitions at different lag days, respectively. | Heat wave definitions, based on three or more consecutive days with both daily minimum and maximum temperatures greater than the 90th percentile, provided the best model fit. The definition based on the 90th percentile of minimum and maximum temperature with a 3-day duration, showed that females and people aged > 55 years old were at higher risks than males and other different age groups to heat wave related mortality. | ACASIS project (http://www.agence-nationale-recherche.fr/Projet-ANR-13-SENV-0007). |
| Fernandez, 2009 | Madrid | Zambia | Daily maximum temperature and rainfall | Cholera | Southern Africa | Poisson autoregressive model controlling for seasonality and trend | A 1°C rise in temperature 6 weeks before the start of a cholera outbreak explained a 5.2% increase in the number of cases. Additionally, a 50 mm increase in rainfall happening 3 weeks before the outbreak explained a 2.5% increase. The attributable risks were 4.9% for temperature and 2.4% for rainfall. The study suggests that if there is an increase in temperature 6 weeks before the rainy season begins, followed by a rise in rainfall 3 weeks later, both exceeding expected levels, there could be an increase in cholera cases within the following 3 weeks. | None reported / none to report |
| Gikungu, 2016 | Kenya | Kenya | Kenya Meteorological Department: International Research Institute for Climate and Society (IRI) Met  Variables: rainfall, NDVI, relative humidity at 06:00 and at 12:00 GMT, maximum and minimum temperatures and SST. | Rift valley fever | East Africa | A dynamic risk model based on historical RVF outbreaks and climate data was developed from a logistic regression model (within the framework of a generalized linear model) | The assessed components of the model accurately predicted already observed RVF events. The Brier score for each of the models (ranging from 0.007 to 0.022) indicated high skill. The coefficient of determination was higher in Garissa (0.66) than in Murang’a (0.21) and Kwale (0.16). | None reported / none to report |
| Kynast-Wolf, 2010 | Germany | Burkina Faso | Mean temperature and rainfall, for the period 1999-2003. Source: meteorological station Dedougou, 50 km from Nouna. | Cardiovascular deaths (CVDs) | West Africa | Verbal autopsy for cause of death. Census for number of residents. Floating absolute risk method (Poisson regression analysis) for effects of each month on specific mortality. Poisson model and a linear regression model for dependance of mortality of CVDs from meteorological variables. Likelihood ratio tests for model validation. | CVDs were the 4th most frequent cause of deaths in adults. Seasonality with temperature was found for all cause of deaths in the adult age group, and for CVDs in the elderly (65+) age group. Mean temperature correlates were found for all-cause mortality for all age groups and for elderly. Maximal temperature correlations were not significant, nor was any temperature correlating with CVDs mortality in any age group. Poisson modelling assumption was tested as appropriate. | Collaborative research grant ‘SFB 544’ of the German Research Foundation (DFG). |
| Grace, 2012 | USA | Kenya | Ground based meteorological stations, remotely sensed data, and a priori knowledge of climatic patterns within that region (sources resulted in interpolated Climate datasets: rainfall and temperature for 1960-1989 and 1960-2009 periods) | Stunting | East Africa | HAZ is a standard measurement of child stunting and was used as a measure of chronic child malnutrition. To determine if child hunger at the micro-level was impacted by climate variables, a multi-level linear regression model with HAZ as the response variable, was constructed | Precipitation level has a significant effect on child stunting. The relationship between temperature and child stunting was not significant. | Famine Early Warning Systems Network (FEWS NET) and US Geographical Services (USGS) |
| Guo, 2023 | China | Multiple | Global One Health Intrinsic Drivers index (GOH-IDI) project is stored in GitHub | One Health | all | This study described Global distribution of GOH-IDI, and analyzed the correlation between GOH-IDI score and Gini coefficient | Low-income countries have the best performance in some secondary indicators, including Non-communicable Diseases and Mental Health and Health risks. Five indicators are not statistically different at each economic level, including Animal Epidemic Disease, Animal Biodiversity, Air Quality and Climate Change, Land Resources and Environmental Biodiversity. | National Key Research and Development Program of China; National Natural Science Foundation of China |
| Hamlet, 2018 | United Kingdom | Multiple | LandScan dataset; (Seasonal air temperature, the enhanced vegetation index (EVI), rainfall; temperature suitability index, the interaction of temperature suitability and rainfall) | Yellow fever (YF) | all | Formulae to determine the basic reproductive number; the temperature suitability index z; the temperature dependent death rate μ(T) contributed to the modelling for temperature dependent mortality | The seasonal model accurately captured both the geographic and temporal heterogeneities in YF transmission (AUC = 0.81) and did not perform significantly worse than the annual model which only captured the geographic distribution. The interaction between temperature suitability and rainfall accounted for much of the occurrence of YF. | Bill & Melinda Gates Foundation |
| Ibekwe, 2019 | Nigeria | Nigeria | Tropical weather conditions | Atopic dermatitis (AD) | West Africa | Univariate analyses and multiple regressions | Patients had a 1.6× odds of presenting with AD in the dry season as compared to wet season. Dry season was associated with higher temperatures and UV index and lower precipitation, humidity and cloud cover. Precipitation (OR: 1.01 p = 0.002), humidity (OR: 1.03 p = 0.015) and cloud cover (OR: 0.92 p = 0.002) exert the most significant effects. Infants had 4.4 times odds of AD than adults (p<0.001). | Self-funded by the authors |
| Jankowska, 2012 | United States | Mali | Temperature and precipitation | Malnutrition | West Africa | Spatial analysis of coupled climate-health dynamics | An additional 6 million people, 1 million children, and heightened risk of malnutrition. | NASA Precipitation Monitoring Mission grant, US Geological Survey’s Geographic Analysis and Monitoring Program |
| Jusot, 2012 | Niamey, Niger | Niger | Minimal and maximal temperature, minimal and maximal relative humidity, wind speed | Influenza | West Africa | Generalized additive model | The majority of influenza cases were detected at low minimal temperatures during the cold season and the rainy season. | None reported / none to report |
| Jutla, 2015 | United States | Zimbabwe | hydroclimatic conditions | Cholera | Southern Africa | Spatial estimation by satellite of precipitation and global gridded air temperature captured sensitivities in hydroclimatic conditions. | Climatic factors in the region were found to be associated with triggering cholera outbreak and are shown to be related to anomalies of temperature and precipitation, validating the hypothesis that poor conditions of sanitation, coupled with elevated temperatures, and followed by heavy rainfall can initiate outbreaks of cholera. | NASA grant |
| Kacem, 2010 | Tunisia | Tunisia | Air pollution data and PE patients from 2 academic hospitals | Pulmonary embolism (PE) | Northern Africa | Data analysis using SPSS Students t-test Pearsons Chi Square test Poisson Distribution | Some environmental parameters may predispose to the onset of idiopathic PE. | None reported / none to report |
| Kapwata, 2018 | South Africa | South Africa | Apparent temperature (AT), household questionnaires | Heat effects | Southern Africa | Regression models | The number of days during which indoor AT categorized as potentially harmful will increase in the future. | South African Medical Research Council Seed Flagship Grant, as well as funds from National Treasury under its Economic Competitiveness and Support Package. |
| Katale, 2023 | Namibia | Namibia | Variable: 2018–2020 climatic dataset Source: Southern African Science Service Centre for Climate Change and Adaptive Land Management (SASSCAL) | Malaria | Southern Africa | Global spatial autocorrelation statistics (Moran’s I) to detect the spatial autocorrelation of malaria cases ; Local Moran statistics used to identify malaria occurrence clusters ; A hierarchical Bayesian CAR model (Besag, York and Mollie’s model “BYM”) to examine climatic factors explaining spatial/temporal variation of malaria incidence rates infection; Using Markov Chain Monte Carlo (MCMC) or Integrated Nested Laplace approximation (INLA) to allocate parameters to cope with their volatility prior to distribution; queens contiguity to create an adjacency matrix and a weight matrix; Global and local spatial autocorrelation to detect global spatial autocorrelation to identify spatial clustering constituencies. A hierarchical Bayesian CAR model (Besag, York and Mollie’s model “BYM”) | Average annual rainfall and maximum temperature have a significant spatial and temporal variation on malaria infection. Annual mean cases of malaria increased by 0.6% for every 1 ◦C and mm increase in annual temperature and rainfall. The three-year average incidence rate (2018–2020) was found to be 6 cases per 1000 population, with the incidence rate being very high in 2018, 12 cases per 1000 population, and decreasing to 1 case in 2019, but unfortunately increasing again in 2020 from 1 case to 4 cases per 1000 population. The Global Moran’s I statistics value was found to be positive: 0.1863 (p - value = 0.0429) with a variance of 0.0159, indicating a spatial autocorrelation in the data at the constituency level where malaria was clustered in these 3 Northern regions of Namibia; The spatial Negative Binomial BYM model results indicate that annual monthly average temperature (mean), annual monthly maximum temperature (mean), annual monthly total rainfall (mean), and annual monthly average wind speed (mean) all had a significant positive effect on annual mean malaria incidence, whereas annual monthly average humidity (mean) and annual average leaf wetness (mean) had a significant negative effect; Strong spatial and temporal heterogeneity distribution of malaria cases (spatial pattern) with high risk in most of the Kavango West and East outskirt constituencies, posterior relative risk (RR: 1.57 to 1.78). | None reported / none to report |
| Katile, 2022 | Mali | Mali | Variable: daily number of rainfall events and cumulative rainfall (mm); minimum and maximum day and night temperatures (◦C); and minimum and maximum day and night relative humidity (%). Mean air pressure (hPa); normalized difference vegetation index; mean wind speed (km/h).  Source: open-access remote sensing data extracted from the NASA Giovanni website; ERA5 database through Google Earth Engine; | Malaria | West Africa | Principal component analysis to correlate meteorological and environmental variables, combined into synthetic indicators (SI), Lag and relationship between the main SIs and malaria incidence determined through a generalized additive model Change-point analysis to determine transmission periods  SatScan method to determine high-risk clusters (hotspots)  Classification and regression tree analysis to rank High-risk clusters (hotspots) according to risk level | Positive and almost linear relationship between the first SI (river flow and height, relative humidity, and rainfall) and malaria incidence non-linear relationship between the second SI (air pressure and temperature) and malaria incidence; The two transmission periods per year: a low transmission period from January to July—corresponding to a persisting transmission during the dry season—and a high transmission period from July to December;  spatial distribution of malaria hotspots varied according to the transmission period. | the French ARTS grant, from the French Research Institute for Development, and by the ‘Dynamique Spatio-temporelle de la Transmission du paludisme dans des Environnements Changeants, Jeunes Equipes associées à IRD (JEAI Dynastec).’ |
| Khalis, 2022 | Morocco | Morocco | Meteorological parameters (average temperature, wind, relative humidity, precipitation, duration of insolation) and air quality parameters (CO, NO2, 03, SO2, PM10) | COVID-19 | Northern Africa | General additive model | Positive associations were observed between COVID-19 and wind above 20 m/s and humidity above 80%. Temperatures above 25 mm were negatively associated with daily cases of COVID-19. PM10 and O3 had a positive effect on the increase in the number of daily confirmed COVID-19 cases, while precipitation had a borderline effect below 25 mm and a negative effect above this value. | Hassan II Academy of Science and Technology, Morocco |
| Komen, 2015 | South Africa | South Africa | Temperature and rainfall (weekly data); source: South Africa Weather Services. | Malaria | Southern Africa | Inverse distance weighted (IDW) method for spatial weighting of malaria records; linear correlation analysis for interdependencies between malaria and climate drivers; multivariate Granger causality test for malaria and combination of temperature and rainfall; Augmented Dickey–Fuller (ADF) and Kwiatkowski, Phillips, Schmidt and Shin(KPSS) stationarity tests for all variables; Autoregressive Distributed Lag (ARDL)–Bounds Test Model for testing causality in non-stationary series | Strong positive correlation of climate variables to malaria cases, with temperature exhibiting a stronger influence as compared to rainfall (correlation coefficients: 0.52 and 0.28, respectively); unidirectional causality from both temperature and rainfall to malaria; non-stationarity found only for temperature; lag length is 3 (months) | FP7 EU project QWeCI (Quantifying Weather and Climate Impacts on health in developing countries) grant no. 243964 |
| Kipruto, 2017 | Kenya | Kenya | Source: International Research Institute / Lamont-Doherty Earth Observation climate database Variables: Maximum temperature, rainfall in four zones, riverine, lowland, mid-altitude and highland | Malaria | East Africa | Negative binomial regression model with lagged climate variables was used to model long-term monthly malaria cases. The seasonal Mann–Kendall trend test was then used to detect overall monotonic trends in malaria cases. | Rainfall at a time lag of 2 months resulted in an increase in malaria transmission across the four zones while an increase in temperature at time lags of 0 and 1 month resulted in an increase in malaria cases in the riverine and highland zones, respectively. | WHO/TDR/IDRC |
| Kirolos, 2021 | UK | Malawi | Source: Global Surface Summary of the Day database  Variables: temperature and rainfall | TB | Southern Africa | Generalized Linear Models (GLMs) using a distributed lag nonlinear framework | TB case notification rate (CNRs) peaks occurred with increasing temperature in September and October before the onset of increased rainfall, and later in the rainy season during January - March, after sustained rainfall. When lag between a change in weather and TB case notifications was accounted for, higher average rainfall was associated with an equivalent six weeks of relatively lower TB notification rates, whereas there were no changes in TB CNR associated with change in average temperature. TB CNRs had a seasonal pattern of two cyclical peaks per year, coinciding with the start and end of the rainy season. | Wellcome Trust Clinical PhD Programme Fellowship National Institute for Health Research UK Government |
| Kitawa, 2023 | Ethiopia | Ethiopia | Source: worldclim.org/data/monthlywth.html and worldclim21.html The average monthly relative humidity is derived from ECMWF Medium-Range Weather Forecasts from ERA Interim global atmospheric reanalysis. Finally, nighttime light (NTL) is obtained from NOAAs (National Centers for Environmental Information), Visible Infrared Imaging Radiometer Suite available at approximately 100 m at the equator. Variables: Monthly temperature (0C) and total precipitation (mm) is extracted from weather and climate data provided at 2.5 min or (∼ 21km^2^) spatial resolution, nighttime light | Malaria | East Africa | Space-time malaria risk mapping | Risk factors like precipitation, temperature, humidity, and nighttime light are significantly associated with malaria with different rates across the districts. | None reported / none to report |
| Kleinschmidt, 2001 | South Africa | South Africa | Spatial distribution of winter climatic conditions, including rainfall and temperature (higher winter rainfall and a higher average maximum temperature) | Malaria | Southern Africa | Spatially adjusted, multiple regression analysis | Malaria incidence was significantly positively associated with higher winter rainfall and a higher average maximum temperature and was significantly negatively associated with increasing distance from water bodies | None reported / none to report |
| Li, 2023 | China | Multiple | Extremely high temperature (EHT) | Macrosomia | Southern Africa and West Africa | Satellite inversion models | Exposure to EHT during pregnancy was significantly associated with a risk of  macrosomia compared with non-exposure maternal, with the RR of  1.507 in 14 countries (95% CI: 1.355, 1.676). Eastern Africa had the  largest RR of 1.372 (95% CI: 1.281, 1.469), while central and southern  Africa had the smallest RR (RR = 1.225, 95% CI: 1.087, 1.381). | Project for Top Disciplinary Talents of Majors in Universities of Anhui Province, Grant/Award Number: gxbjZD09 |
| Mabaso, 2006 | South Africa, Switzerland, Zimbabwe | Zimbabwe | Variable: mean annual values of rainfall, vapor pressure, minimum, maximum and mean temperature;  Source: the climate research unit (CRU) climate surfaces derived from interpolated weather station data as a function of latitude, longitude, and elevation using thin-plate splines | Malaria | Southern Africa | Bayesian negative binomial models for spatio-temporal analysis of the relationship between annual malaria incidence and selected climatic covariates; Spatial correlation was incorporated by assuming a conditional autoregressive (CAR) process in the random effects. A first order autoregressive process was applied for temporal random effects; Bayesian negative binomial models were fitted in WinBUGS  to examine the association between inter-annual variation in malaria incidence and a combination of climatic covariates selected from the preliminary analysis; A preliminary negative binomial regression analysis  carried out in STATA 9.0 (Stata Corp., College Station, TX, USA) to assess the relationship between annual malaria incidence and annual values of each climatic covariate; Markov Chain Monte Carlo simulation (MCMC) was applied to estimate model parameters. Deviance Information Criterion (DIC) was used for the comparison of model fit. Model estimates were exponentiated to represent incidence rate ratios (IRR), that is, per unit change in incidence for each | Annual mean values of average temperature, rainfall and vapor pressure were strong positive predictors of increased annual incidence whereas maximum and minimum temperature had the opposite effects. High annual malaria incidence coincides with high rainfall and relatively warm conditions while low incidence years coincide only with low rainfall. Temperature derived covariates seem to be important only in the presence of sufficient rainfall. The intensity and timing of the seasonal peak in each year appears to follow variability in rainfall. In the bivariate analysis, all selected covariates showed a significant relationship (P < 0.001) with malaria incidence, mean annual temperature, rainfall, vapor pressure and NDVI were strong positive predictors of increased annual incidence rate. | Rudolf Geigy Stiftung Zu Gunsten des Schweizerischen Tropeninstituts and partially by the Swiss National Science Foundation Project 3252B0-102136/1 |
| Mabaso, 2005 | South Africa | Zimbabwe | Source: Climate Research Unit (CRU) interpolated climate surfaces with a 0.5 · 0.5 grid resolution  Variables: Mean monthly values of rainfall, vapor pressure, temperature as well as maximum and minimum temperature | Malaria | Southern Africa | Spatial-temporal regression model within a Bayesian framework | Combinations of mean monthly temperature (range 28–32 °C), maximum temperature (24–28 °C) and high rainfall provide suitable conditions for seasonal transmission. High monthly maximum and mean monthly minimum temperatures limit months of high transmission. | This work was funded partly by the Rudolf Geigy Stiftung Zu Gunsten des Schweizerischen Tropeninstituts and the Swiss National Science Foundation Project 3252B0-102136/1. |
| Makunyane, 2023 | South Africa | South Africa | Temperature variability Meteorological data were obtained from the South African Weather Service | Cardiovascular (CVD) and respiratory disease (RD) | Southern Africa | Quasi-Poisson regression model | The study found a strong link between temperature variability (TV) and hospital admissions for CVD and RD. This association remained significant even after considering factors like daily temperature and humidity. TV had the most impact on hospitalizations when measured over short periods, particularly affecting older individuals for RD and middle-aged adults for CVD. Men were more likely to be hospitalized than women. | The South African National Research Foundation grant number: 111614, and from the Department of Science and Innovation through the iDEWS Project and ACCESS program hosted by the Council for Scientific and Industrial Research partnering with the South African Weather Services. |
| Malone, 2994 | USA | Egypt | Source: NOAA satellite data; Temperature, minimum, maximum and diurnal temperature range | Schistosomiasis | Northern Africa | Spearman correlation coefficient | Some evidence of a relation between schistosomiasis and diurnal temperature range was found. | National Institutes of Health, USAID, Binational Fulbright Commission (Egypt) |
| Kulkarni, 2016 | Canada | Tanzania | 2004 and 2014-2015: land surface temperature, at 500m grid source: MODIS 2004 and 2014: temperature and rainfall records. Source: Kilimanjaro International Airport weather station. | Vector | East Africa | Environmental niche modeling, using Maximum Entropy (maxent) software, correlation analysis between variables used. Land cover was used as a categorical variable and temperature, vegetation indices, elevation, and human population density as continuous variables. | Climate, land use, and population changes may have an important influence on the distribution of *An. arabiensis.* The vector has gone through noticeable range expansion at higher altitudes and range depletion at lower altitudes. Difference found of 2 °C and 30 persons/km^2^ between years 2004 and 2014, which may be factors that influenced found change. Increase in human population may have led to the increase in deforestation that led to an increase in the number of vectors. | Grand Challenges Canada grant no. S6-0476-01-10 and a University of Ottawa Faculty of Medicine operating grant. |
| Matthew Olanyinan, 2020 | Nigeria | Nigeria | Variable: daily records of rainfall, relative humidity, wind speed and temperature (minimum and maximum) as well as diurnal temperature; Source: Climate Observatory located within the Space Applications and Environmental Laboratory (SPAEL), Institute of Ecology and Environmental Studies | Malaria | West Africa | A fuzzy logic suitability (FLS) model to determine the suitability conditions for malaria transmission across seasons. Conducting multivariate regression analysis and lag correlation for up to 4 months. Calculation of linear trends using a least square fit to identify significant or non-significant trends. Application of multiple linear regression models and lag correlation analysis. | Mosquitoes could survive all-year round with p values ranging between 0.40 and 0.96 under the prevailing mean climate. Rainfall was the best predictor (r = 0.7, R2 = 0.448, p < 0.05) and no significant time-delay effect was noticed between climatic variables and malaria occurrence except for wind speed at 1- month lag. About 61% (multiple R2 = 0.613 at p = 0.1) of monthly variations in reported malaria cases accounted for by climate variability; Reported malaria cases were generally higher in wet months than dry months; Good positive agreements (0.669 ≤ r ≤ 0.692 at p < 0.05) between the reported malaria cases and monthly rainfall and FLS conditions were found; a positive significant correlation (r = 0.7, p < 0.05) between malaria caseloads and wind speed was observed at a 1-month lag; About 45% of variations in malaria cases were accounted for by rainfall (R2 = 0.448) and 48% by FLS (R2 = 0.478)—both significant at p < 0.05. | None reported / none to report |
| Mazamay, 2020 | Democratic Republic of Congo | Democratic Republic of Congo | Source: WorldClim—Global Climate Data; Variables: Average monthly rainfall and temperature | Meningitis | Central Africa | Generalized Linear and Mixed Models | The regions with warm temperature and a population with a low index of economic well-being (IEW) constitute the perfect conditions for the development of meningitis in DRC. | The NGO « Organisation pour le Developpement Durable de Mai-ndombe » (NGOODDM), the URF-ECMI (Training and Research Unit in Ecology of infectious diseases, Medicine Faculty, University of Kinshasa, DRC) and IRD/MIVEGEC, UMR CNRS IRD UM, Montpellier, France. Jean-Francois Guegan is supported by an “Investissement d’Avenir” grant managed by Agence Nationale de la Recherche (LABEX CEBA:ANR-10-LABX-25-01), and is also supported by Institut de recherche pour le développement (IRD), Institut national de recherche pour l’agriculture, l’alimentation et l’environnement (INRAE), Université´ de Montpellier and Ecole des Hautes Etudes en Sante´ Publique (EHESP). |
| McLaughlin, 2023 | United Kingdom | Malawi | Temperature variability | Malnutrition | Southern Africa | Fixed-effects model to household panel data | The study showed that fluctuations in weather, particularly rainfall and temperature, have implications for food and nutrition security in Malawi. It anticipates that with climate change, leading to more frequent and severe weather variations, the prevalence of micronutrient deficiencies in the country will likely rise. The study underscores the value of household consumption and expenditure surveys (HCES) as vital resources for assessing food and nutrition security, particularly in developing countries where more precise data collection methods may be impractical. | Department for the Economy (DfE), Northern Ireland Research Studentship. |
| Mendelsohn, 2008 | South Africa | South Africa | Precipitation, sea surface temperature and chlorophyll-a data | Cholera | Southern Africa | Observational: Identification of trends, time lags and coefficients of determination were used to characterize and measure relationships between climate and epidemiological data. | Environmental factors played a significant role in a cholera outbreak in KZN, SA. Rainfall and sea surface temperature were strong drivers of the epidemic, while chlorophyll-a had a moderate impact, and sea surface height had a weaker effect. These findings suggest a possible coastal transmission hypothesis linked to heavy rainfall. | None reported / none to report |
| Mrema, 2012 | Tanzania | Tanzania | Source: Tanzania Meteorological Authority; temperature and rainfall | Mortality | East Africa | Time series Poisson regression model with distributed lag model | Rainfall was associated in the 0-4 years age group in both short and long lag times with an overall increase in mortality risk for a 10 mm rise in rainfall. Monthly average temperature had a stronger association with death in all ages while mortality increased with falling monthly temperature. | None |
| Mukhtar, 2019 | South Africa | South Sudan | Sources: European Centre for Medium-Range Weather Forecasts Regional Meteorological Service | Malaria | Northern Africa | Bayesian framework using Bayesian Markov Chain Monte Carlo | Malaria infection peaks are associated with increases in temperature and rainfall. | None reported / none to report |
| N’gattia, 2016 | Cote d’Ivoire | Cote d’Ivoire | Weekly cumulative rainfall, average weekly relative humidity, and average weekly ambient temperature were obtained from the database of the Airport Operating Development Aviation and Meteorology Company (SODEXAM). | Influenza | West Africa | Autoregressive integrated moving average models (ARIMA) | Significant cross-correlation was observed between the weekly incidence of influenza cases and weekly cumulated rainfalls at lags (current and prior weeks) Epidemiological Week 0 (r=0.187) and Epidemiological Week−5 (0.175) and the average weekly relative humidity. | CDC-Atlanta Influenza Division |
| Nigussie, 2022 | Ethiopia | Ethiopia | Ethiopian Metrology Agency (EMA). The satellite and model based reanalyzed data were downloaded from the US National Oceanic and Atmospheric Administration (NOAA), Giovanni open data portal of NASA, Climate Hazard Center InfraRed Precipitation with Station data (CHRIPS), and database of global climate & weather data (WorldClim.com) total rainfall, land surface temperature, soil moisture, relative humidity, percentage coverage of at least one long-lasting insecticide net, and the minimum and maximum temperature. | Malaria | East Africa | Poisson regression model | Monthly minimum temperature had an indirect effect on the malaria incidence (95% CI: − 0.0349, − 0.0097) and showed that the risk of malaria decreased by 2.2% for a 1 ◦C increment in the average minimum temperature of districts by holding the other covariates constant. However, total monthly rainfall directly affected the risk of malaria (95% CI: 0.0005, 0.0012), which increased by 8% for a 100-mm increment in the total monthly rainfall of districts. | None reported / none to report |
| Njifon, 2019 | Cameroon | Cameroon | Cumulative rainfall, average monthly ambient temperature, monthly relative humidity. | Influenza | Central Africa | Data were analyzed using EviEws software version 9. A VECM (vector error correction model) was used to estimate the model which best explains the variations in influenza activity. | Average RH was the only meteorological variable to show a significant and positive association to overall influenza activity and to influenza A activity. Accumulated rainfall and mean temperature were not associated with influenza activity in the North region. | US Department of Health and Human Services via international network of Pasteur Institutes |
| Nkurunziza, 2011 | Burundi | Burundi | Rainfall, maximum temperature, minimum temperature, maximum humidity and minimum humidity. | Malaria | central Africa | Used Generalized Additive Mixed Model (GAMM) | The results of the GAMs show that an increase in the maximum temperature will cause an increase in minimum temperature. The increase in the latter will result in a decreasing maximum humidity, leading to a decrease in rainfall. These results suggest that an increased temperature will result in a shortening of the life span of mosquitoes (due to decreasing humidity) and decrease in the capacity of larva production and maturation (due to decreasing rainfall). The increase in temperature will not result in an increased malaria transmission in Burundi. | Austrian agency for international cooperation in education and research (ÖAD) |
| Oheneba‑Dornyo, 2022 | Ghana | Ghana | Temperature, rainfall | Malaria | West Africa | Panel data analysis, using two modelling approaches were used: fixed effects models (FEM) and random effects models (REM). | Malaria prevalence gradually declined from 2012 to 2017.Periods of high and low malaria prevalence coincide with the wet and dry seasons, respectively. There is a statistically significant association between malaria prevalence and climatic variation using fixed-effect modelling. | The study was undertaken with a Master’s student supported by funding of the University of Exeter and the Commonwealth Shared Scholarship Commission. |
| Omonijo, 2013 | Nigeria | Nigeria | Air temperature, relative humidity, wind speed, solar radiation | Heat related physiological effects | West Africa | RayMan model as an analytical tool to compute physiologically equivalent temperature (PET) in order to assess thermo-physiological thresholds. | 60 % of the total study period (1998–2008) fell under physiological stress level of moderate heat stress. | Partially funded by the African Doctoral Dissertation Research Fellowship offered by the African Population and Health Research Center (APHRC) in partnership with the International Development Research Centre (IDRC). |
| Omonijo, 2012 | Germany | Nigeria | Meteorological variables: air temperature, relative humidity, wind speed, solar radiation, | Measles | West Africa | Stepwise multiple regression; Analysis of variance (ANOVA), including F-test, P-value  of the models and determination coefficient R2, which measures the goodness-of-fit of regression model at a confidence limit of 95%, was performed. | The highest transmission of measles is between the months of January and May when the meteorological/ biometeorological parameters are high. During this period, the values of PET ranged between 35°C and 38°C, while that of Universal Therman Climate Index (UCTI) is between 34°C and 37°C, mean radiant temperature (Tmrt) is between 41°C and 44°C and air temperature is 32°C–34°C in the derived savannah zone of Ondo State. In the humid forest zone of Ondo State, the value of PET  is between 32°C and 35°C, UTCI is 32°C–34°C, Tmrt is between 37°C and 40°C, and solar radiation values ranged from 153 Wm–2 to 166 Wm–2 during high transmission of measles in this zone. | Alexander von Humboldt Foundation for the award of an International Climate Protection Fellowship. African Population and Health Research Center (APHRC) and the International Development Research Center (IDRC) |
| Ototo, 2022 | Kenya | Transboundary park-Kenya, Tanzania and Uganda | Rainfall and temperature | Malaria | Southern Africa | The vector autoregressive moving average processes model, VARMAX is then used to forecast malaria and anemia responses to rainfall and temperatures projected with an ensemble of eight General Circulation Models (GCMs) for climate change scenarios defined by three Representative Concentration Pathways (RCPs 2.6, 4.5 and 8.5). | Historical malaria cases are positively and linearly related to the 3–6-month running means of monthly rainfall and maximum temperature. The short rains (OND), wet season (MAM) temperatures and clinical malaria cases will likely increase in the Lake Victoria Basin. | Open Access funding enabled and organized by Projekt DEAL. International Development Research Center/ Department for International Development IDRC/DFID (CCAA, Project ID: 104707-001). German Research Foundation (DFG Grant # 257734638). This research was supported with funds from USAID/Kenya and East Africa Planning for Resilience in East Africa through Policy, Adaptation, Research and Economic Development (PREPARED). This project has received funding from the European Union’s Horizon 2020 research and innovation programme under Grant Agreement No. 641918. |
| Ouattara, 2022 | Burkina Faso | Burkina Faso | Maximum and minimum temperature, relative humidity, and wind speed. | Dengue fever | West Africa | General Additive Model | Maximum and minimum temperature, relative humidity, and wind speed have a significant non-linear effect on dengue cases in the region with 83% of case variance explained. | None reported / none to report |
| Panzi, 2022 | Democratic Republic of Congo | Democratic Republic of Congo | Source: Mettelsat database speed  Variables: total rainfall quantity, temperature, wind, total evaporation under shelter, and average relative humidity | Malaria | Central Africa | Generalized Poisson Regression | When total evaporation under shelter increases by one unit, the probability of malaria decreases by 2% (p<0.0001). When the average relative soil moisture increased by one percentage point, the risk of malaria morbidity decreased by 0.42% (p<0.0001). Adjusting for, other factors, one-day increase in rainfall resulted in a 7% increase in malaria cases. This relationship was statistically significant (p<0.0001). For each increase in maximum temperature of one degree Celsius, the risk of malaria infestation  increased by 1.08 times cases (p < 0.0001). When the average wind speed at 2 m above the ground increased by one meter per second, the risk of a malaria episode decreased by 18%. This relationship was statistically significant (p < 0.0001). | DELTAS Africa Initiative SSACAB [grant #DEL-15005]. The DELTAS Africa Initiative is an independent funding scheme of the African Academy of Sciences (AAS)’s Alliance for Accelerating Excellence in Science in Africa (AESA) and supported by the New Partnership for Africa’s Development Planning and Coordinating Agency (NEPAD Agency) with funding from the Wellcome Trust [grant #107754/Z/15/Z] and the UK government. |
| Pascual, 2008 | USA | Kenya | Source: Kenyan Meteorological Department  Variable: rainfall | Malaria | East Africa | Time-series epidemiological model (time-series susceptible infected–recovered) and a statistical approach specifically developed for non-stationary patterns. | The short rains appear associated with the small peak of malaria at the beginning of the following year. This is important because the number of cases in this first outbreak show themselves a significant correlation with the number of cases later in the year. Thus, anomalous rainfall in the short rains would display a ripple effect and affect the total number of cases in the following year. | National Center for Ecological Analysis and Synthesis (NCEAS), a center funded by NSF and UC Santa Barbara. Support was also provided by a Centennial Fellowship by the James S. McDonnell Foundation in Global and Complex Systems, and by joint funding from the National Science Foundation–National Institutes of Health (Ecology of Infectious Diseases Grant EF 0430 120) and the National Oceanic and Atmospheric Administration (Oceans and Health Grant NA 040 AR 460019). |
| Paz, 2009 | Isreal | South- Eastern Africa | Annual variability of air temperature and sea surface temperature at regional and hemispheric scales | Cholera | South-Eastern Africa | Poisson Regression Model | The study found that there was a significant increase in cholera cases over time and the annual average temperature and sea surface temperatures in the area, as well as unusual temperature patterns on a larger scale, had a statistically significant effect on cholera prevalence during the study. | None reported/ none to report |
| Pedder, 2021 | United Kingdom | South Africa | Temperature-lag; Relative humidity -lag | Pneumonia | Southern Africa | A time-varying distributed lag non-linear model was used to estimate temperature-lag and relative humidity-lag pneumonia relationships. | Mean temperature, relative humidity and diurnal temperature range were all significantly associated with pneumonia admissions. Cumulatively across the 21-day period, higher mean daily temperature (30 °C relative to 21 °C) was most strongly associated with a decreased rate of hospital admissions (relative rate ratios (RR): 0.34, 95% confidence interval (CI): 0.14–0.82), whereas results were suggestive of lower mean daily temperature (12 °C relative to 21 °C) being associated with an increased rate of admissions (RR: 1.27, 95%CI: 0.75–2.16). Higher relative humidity (>80%) was associated with fewer hospital admissions while low relative humidity (<30%) was associated with increased admissions. A proportion of pneumonia admissions were attributable to changes in meteorological variables, and our results indicate that even small shifts in their distributions (e.g., due to climate change) could lead to substantial changes in their burden. | SATREPS (Science and Technology Research Partnership for Sustainable Development) Program of JICA (JAPAN International Cooperation Agency)/AMED (Japan Agency for Medical Research and Development) in Japan, the ACCESS (Applied Centre for Climate and Earth Systems Science) program of NRF (National Research Foundation) and DST (Department of Science and Technology in South Africa) and from the Quality Related Global Challenges Research Fund of the University of Bristol. Also, the South African Medical Research Council. |
| Phiri, 2021 | Zambia | Zambia | Source: United States Geological Survey Variable: topographic and climatic factors, | COVID-19 | Southern Africa | Decision or classification trees | The results showed that the distribution of COVID-19 cases in Zambia was significantly influenced by the socioeconomic factors compared to environmental factors. | Japan Society for the Promotion of Science under Grant-in-Aid for Scientific Research |
| Randell, 2020 | USA | Ethiopia | Source: Variables: rainfall and temperature | Malnutrition | East Africa | Multivariate regression models | Greater rainfall during the rainy seasons in early life is associated with greater height for age. In addition, higher temperatures in utero, particularly during the first and third trimesters, and more rainfall during the third trimester, are positively associated with severe stunting, though stunting decreases with temperature in early life. | None reported / none to report |
| Sehlabana, 2020 | South Africa | South Africa | Source : Ecoverb; variables, rainfall, night temperature, day temperature, | Malaria | Southern Africa | Bayesian and classical methods of estimation | The Bayesian method appeared more robust than the classical method in analyzing malaria incidence in Limpopo Province. The classical method identified rainfall and temperature during the night to be significant predictors of malaria incidence in Mopani, Vhembe and Waterberg districts. However, the Bayesian method found rainfall, normalized difference vegetation index, elevation, temperatures during the day and night to be the significant predictors of malaria incidence in Mopani, Sekhukhune and Vhembe districts of Limpopo Province. Both methods affirmed that Vhembe district is more susceptible to malaria incidence, followed by Mopani district. | None reported / none to report |
| Seid, 2014 | Ethiopia | Ethiopia | Source : National Meteorology Service Agency; 20 consecutive years data derived from more than 100 meteorological stations distributed around the country. Variables: annual average temperatures and annual rainfall | Cutaneous leishmaniasis (CL) | East Africa | Risk model based on environmental factors using geographical information systems (GIS), statistical analysis and modelling | When estimating risk from the viewpoint of geographical surface, slope, elevation and annual rainfall were found to be good predictors of CL presence based on both probabilistic and weighted overlay approaches. However, when considering Ethiopia as whole, a minor difference was observed between the two methods with the probabilistic technique giving a 22.5% estimate, while that of weighted overlay approach was 19.5%. Calculating the population according to the land surface estimated by the latter method, the total Ethiopian population at risk for CL was estimated at 28, 955, 035, mainly including people in the highlands of the regional states of Amhara, Oromia, Tigray and the Southern Nations, Nationalities and Peoples’ Region, one of the nine ethnic divisions in Ethiopia. The environmental risk model provided an overall prediction accuracy of 90.4%. | The World Health Organization, Swedish and Norwegian Development Agencies (SIDA and NORAD) |
| Seidu, 2013 | Norway | Ghana | Source : Ghana Meteorological local station in Tamale; variables: Bi-Weekly Temperature(Max and Min)Bi-Weekly Rainfall (Max and Min) | Diarrhea | West Africa | Autoregressive Poisson regression models | Maximum rainfall events in the same bi-week increased the risk of diarrhea in the sludge (relative risk, RR: 1.034; confidence interval, CI: 1.02–1.05) and non-sludge (RR: 1.003; CI: 0.99–1.01) communities. However, this was not significant in the non-sludge communities (p> 0.05). Minimum rainfall occurring in the same biweekly decreased the risk of diarrhea in both communities. Maximum temperature decreased the risk of diarrhea in the sludge communities (RR: 0.50; CI: 0.38–0.65) but increased the risk in the non-sludge communities (RR: 1.19 CI: 1.02–1.40). Minimum temperature increased diarrhea disease risk (RR: 3.50; CI: 2.10–5.80) in the sludge communities but decreased the risk (RR: 0.70; CI: 0.54–0.84) in the non-sludge communities. | None reported / none to report |
| Selmane, 2015 | Algeria | Algeria | National Meteorological Office Temperature (T)  Relative Humidity (RH)  Wind Speed (W), Evaporation (E) Precipitation (P) | Cutaneous leishmaniasis (CL) | Northern Africa | Time series analysis based on the Box-Jenkins method to fit an autoregressive moving average (ARMA- 3,3) model incorporating climate factors to the monthly recorded cutaneous leishmaniasis (CL) cases in Biskara province. | An ARMA (3,3) model incorporating temperature at a lag of 5 months and relative humidity was appropriate for forecasting the monthly data for CL between 2000 and 2009 in Biskara province. Temperature had higher effect followed by relative humidity. The model was used for predicting monthly CL cases from January 2010 to December 2014; the predictions matched the recorded data. |  |
| Semakula, 2017 | China | Sub-Saharan Africa | Source : the 21 CMIP5 models; variables: temperature, rainfall | Malaria | Southern Africa | Authors combined Geographical Information System (GIS) and Bayesian belief networks (BBN) to generate GIS-BBN models | The model on which projections were made has an accuracy of 80.65% to predict the high, medium, low and no malaria prevalence categories correctly. | Programme of Introducing Talents of Discipline to Universities (B13012) |
| Shimaponda-Mataa, 2017 | Zambia | Zambia | Source: Moderate Resolution Imaging Spectroradiometer(MODIS) Variable: Day and night land surface temperature (LST) | Malaria | Southern Africa | Structured Additive Semiparametric Poisson regression model | Malaria incidence had a quadratic relationship with rainfall and maximum temperature, incidence and minimum temperature were non-linear. | None reported / none to report |
| Sewe, 2015 | Sweden | Kenya | Temperature and rainfall. | Malaria/ anemia | East Africa | A general additive model with a Poisson link function | Deaths from malaria or anemia were linked to changes in temperature and rainfall, with effects sometimes seen up to 16 weeks later. These findings match what we know about how these diseases are affected by weather. Knowing the strength of these relationships over longer periods can help develop forecasts that predict high transmission periods up to 16 weeks in advance. This could help communities prepare better for malaria outbreaks. | INDEPTH Network. The Swedish Council for Working Life and Social Research |
| Sewe,2016 | Sweden | Kenya | Temperature and rainfall. | Malaria | East Africa | Distributed Lag Non-Linear Modelling approach. | Higher temperatures had different effects depending on the area, while rainfall consistently increased the risk of malaria deaths when weekly precipitation exceeded 20 mm, peaking at 80 mm. The vegetation index also played a role, with increased mortality risk linked to values between 0.3 and 0.4 at shorter time delays. Overall, the study suggests that rainfall is the most reliable predictor of malaria transmission in these regions of Western Kenya. | President's Emergency Plan for AIDS Relief (PEPFAR) and the President's Malaria Initiative (PMI) HHS/Centers for Disease Control and Prevention (CDC), Center for Global Health (CGH), Division of Global HIV/AIDS (DGHA) and Division of Parasitic Diseases and Malaria (DPDM). |
| Sewe, 2017 | Kenya | Kenya | Seasonal Transmission | Malaria | East Africa | General additive model and GAMBOOST model | Hospital admission data for children under five years old confirmed malaria at Siaya district hospital in Western Kenya. There was a total of 8,476 confirmed malaria admissions. The peak of the malaria season changed over time, and malaria admissions decreased gradually. A model called GAMBOOST was most accurate in predicting malaria outbreaks one month in advance during both training and testing. | Swedish Council for Working Life and Social Research |
| Shah, 2019 | United States of America | Kenya | Temperature and rainfall | Malaria | East Africa | Linear mixed model to assess relationships | Malaria rates varied across different clinic sites in Kenya, with the highest rates found in rural areas. Cooler temperatures were observed in Western sites, while warmer temperatures were noted in coastal sites. After considering factors like socioeconomic status, age, and bed net use, the study found that malaria rates peaked around 25°C across all sites, aligning with predictions. This suggests that there is an optimal temperature for malaria transmission, which may be lower than previously thought. As temperatures rise due to climate change, malaria could spread to cooler regions while declining in already warm areas. | National Science Foundation (NSF) grants DEB-1518681 and DEB-1640780 Stanford Woods Institute for the Environment Stanford Center for Innovation in Global Health |
| Sewe, 2018 | Sweden | Kenya and Burkina Faso | daily maximum temperature | Years of life lost | East Africa | A distributed lag nonlinear model | Years of life lost (YLL) increases as temperatures rise. In hot areas like Burkina Faso, YLL consistently rises with higher temperatures. In Nairobi, both high and low temperatures are associated with more YLL. The findings suggest that both high and low temperatures contribute to YLL across countries of different income levels. Using comprehensive indicators like YLL could help guide policies and health adaptation measures related to temperature impacts on health. | Graduate School in Population Dynamics and Public Policy, Umea University Virginia G. Piper Health Policy Informatics Initiative at Arizona State University Klaus-Tschira Stiftung gGmbH Swedish International Development Cooperation Agency (SIDA) William and Flora Hewlett Foundation |
| Siraj, 2015 | United States of America | Ethiopia | Variable: Monthly mean temperature and rainfall from meteorological stations; Surface Temperature (SST) anomalies; sea | Malaria | East Africa | Generalized linear mixed model | Rainfall and mean temperatures in December–February were significantly associated with January, February, May and April malaria cases. Malaria cases in the main transmission season (September, October, November and December) were not significantly associated with rainfall or with population density. Mean temperature was significant in both seasons. | WHO (RBM/WHO) |
| Sissoko, 2017 | Mali | Mali | Source: Rural Economic Institute; Variables: temperature (t1M); minimum inside temperature (t1m); maximum ground temperature (t2M); minimum ground temperature (t2m); temperature at 10 cm (t10); temperature at 20 cm (t20); outside extreme humidity (hx); relative humidity (hr); evaporation using an experimental plug (evg); evaporation using an experimental pan (evn); cumulated rainfall (r); number of rainfall events (nre); sunlight (sl); tension value (tv); wind speed (ws); river height (rvh). | Malaria | West Africa | Principal Components Analysis with lags assessed by cross-correlation function estimations | The main meteorological factor associated with malaria was a combination of evaporation, humidity and rainfall, with a lag of 3 months. The relationship between combined temperature factors showed a linear impact until reaching high temperatures limiting malaria incidence, with a lag of 3.25 months. Height and variation of the river were related to malaria incidence (respectively 6-week lag and no lag). | African Monsoon Multidisciplinary Analysis and by the Prospective & Cooperation NGO. |
| Siya, 2020 | Uganda | Uganda | Source: Not stated Variables: Rainfall and temperature parameters (maximum and minimum) | Malaria | East Africa | Time series | Rainfall and vegetation played a significant role in influencing malaria burdens. | None reported / none to report |
| Sy, 2022 | Senegal | Senegal | Source: Weather Stations Variables: maximum, average and minimum temperatures | Heat effects | West Africa | Logistic regression models | Increase in consultations for climate sensitive diseases at health facilities during heat waves episodes. | Climate Research for Development (CR4D) from the African Academy of Sciences (AAS), the United Nations Economic Commission for Africa (UNECA) and the United Kingdom Aid (UKAID) through the Centre for Ecological Monitoring (CSE) |
| Talla, 2014 | Senegal | Senegal | Source: Weather stations Variable: rainfall, relative humidity, NDVI and temperature | Vector | West Africa | Mixed Bayesian statistical model | The rainfall and minimum temperature were positively correlated with the abundance of *Cx*. *poicilipes*, whereas the maximum temperature had negative effects. The rainfall was negatively correlated with the abundance of *Ae. vexans*. | None reported / none to report |
| Tawiah, 2023 | Ghana | Ghana | Monthly average rainfall, humidity, and temperature | Malaria | West Africa | Multiple regression analyses | The number of confirmed malaria cases increased with increasing rainfall. Increases in temperature reduced the number of malaria cases. | None reported / none to report |
| Taye, 2015 | Ethiopia | Ethiopia | Source: Weather stations  Variables: Monthly maximum and minimum temperature, relative humidity and rainfall | Malaria | East Africa | Generalized Linear Models (GLM) | Malaria prevalence is positively associated with increased rainfall and minimum temperature but negatively associated with maximum temperature. Projections of malaria prevalence showed an increasing trend over the coming years. | World Health Organization (WHO) |
| Teklehaimanot, 2004 | United States of America | Ethiopia | Daily meteorological data (minimum and maximum temperature and rainfall) recorded at the local weather stations | Malaria | East Africa | Robust Poisson regression | Rainfall is identified as a significant factor associated with malaria cases in both hot and cold districts, with a lagged effect observed. This lag is notably shorter in hotter districts. The impact of rainfall on malaria follows a linear pattern, with saturation effects observed at higher levels of rainfall. In the examined Ethiopian districts, it is noted that weather-based predictors of malaria incidence are more applicable in rural settings compared to urban environments. These insights underscore the importance of considering such factors in the development of an effective early warning system for malaria. | Fogarty International Center (FIC) of the National Institutes of Health (NIH) (grant number 5D43TW000918). Financial support for data collection was provided by World Health Organization/RBM. ML thanks the Ellison Medical Foundation for support of this research. |
| Thiede, 2020 | United States of America | Sub-Saharan Africa | High temperatures and rainfall | Malnutrition | Southern Africa | Linear regression model | Temperature and rainfall predict child weight-for-height ratios, with above-average temperatures and below-average precipitation linked to reduced weight. High temperatures increase the risk of wasting, particularly affecting children at the lower end of the weight distribution. Climate effects show no substantial differences by child sex, number of habitants, young children, or maternal education. Urban children experience larger reductions in wasting risk during below-average temperatures compared to rural children. However, both groups face similar increases in wasting risk during high-temperature spells. | Population  Research Institute at the Pennsylvania State University, which is partly  funded through the Eunice Kennedy Shriver National Institute of Child Health and Human Development (P2CHD041025). The USDA National Institute of Food and Agriculture and Multistate Research Project #PEN04623 (Accession #1013257). |
| Tompkins, 2019 | Italy | Uganda | Forecasted temperature and rainfall | Malaria | East Africa | Mathematical models which account for nonlinear relationships between temperature, rainfall and malaria | The Malaria Early Warning System was able to predict seasonal trends in case anomalies showing that the forecasting system was able to predict temporal variations in malaria cases. | EUFP7 National; Institute for Health Research (NIHR); Health Protection Research Unit in Emergency Preparedness and Response at King's College London. |
| Lee, 2023 | Switzerland | South Africa | Daily maximum and minimum temperature, precipitation, and relative humidity at 8:00,14:00, and 20:00 for the period 2010-2019.  Source: South African Weather Service (SAWS) ([www.weathersa.co.za](http://www.weathersa.co.za)) for weather stations in the Cape Town | Diarrhea | Southern Africa | Negative binomial regression model with the log link function; Lagged Pearson’s residual function; Modelling was done only for disease surge season (November to May). Framework method for analysis of the stakeholder interviews. | Numbers of diarrheal cases decreased during the period 2010-2019, in comparison to the previous decade. Maximum temperature and relative humidity at 8:00 are significantly positively associated with incidence of diarrheal disease with dehydration, in the analysis with no lag. This signifies an increase in diarrheal disease with dehydration of 7% for each 1 ˚C increase and 3% increase for each 1% increase in relative humidity. A similar trend was shown in the model with 1 month lag, where maximal temperature and humidity significantly correlate with the disease. Time lags up to 1 month are probably the most plausible ones for correlations between climate and the disease. | University of Basel, South African–Swiss Bilateral SARChI Chair in Global Environmental Health of Mohamed Aqiel Dalvie, the Centre for Environmental and Occupational Health Research, the University of Cape Town, and Martin Röösli, the Swiss Tropical and Public Health Institute - this chair was formed in 2015 with funding sources from the South Africa National Research Foundation (NRF) SARChI (Grant number 94883), Swiss State Secretariat for Education, Research and Innovation, University of Basel and the Swiss TPH. |
| Walker, 2013 | UK | Kenya | Source: Kilifi Institute of Agriculture weather station Variable: rainfall, minimum and maximum temperature | Vector | East Africa | Polynomial distributed lag generalized linear mixed models | Anopheline density was positively and significantly associated with amount of rainfall between 4 to 47 days, negatively and significantly associated with maximum daily temperature between 5 and 35 days, and positively and significantly associated with maximum daily temperature between 29 and 48 days in the past (depending on Anopheles species). | Wellcome Trust, Biology and Biotechnology Scientific Research Council, National Institutes of Health, European Commission FP7 Collaborative project |
| Wu, 2022 | Czech | Global | Temperature variability | Mortality | Africa (global) | Three-staged meta-analytical approach to assess global temperature variability related mortality: Daily time series of 750 locations for location-specific temperature variability related-mortality; Multivariate meta-regression model to estimate grid-specific temperature variability and percentage excess in mortality and excess mortality rates were calculated. | There was an increasing trend in temperature variability at the global level from 2000 to 2019. Globally, 1,753,392 deaths (95% CI 1 159 901–2 357 718) were associated with temperature variability per year, accounting for 3·4% (2·2–4·6) of all deaths. Most of Asia, Australia, and New Zealand were observed to have a higher percentage excess in mortality than the global mean. Globally, the percentage excess in mortality increased by about 4·6% (3·7–5·3) per decade. The largest increase occurred in Australia and New Zealand (7·3%, 95% CI 4·3 – 10·4), followed by Europe (4·4%, 2·2–5·6) and Africa (3·3%, 1·9–4·6). | This study was supported by the Australian Research Council (DP210102076) and the Australian National Health and Medical Research Council (APP2000581). Authors were supported by the China Scholarship Council (number 202006010044). Emerging Leader Fellowship of the Australian National Health and Medical Research Council (number APP2009866). The Program of Qilu Young Scholars of Shandong University, Jinan, China. China Scholarship Council (number 202006010043). The Czech Science Foundation (project number 20–28560S). The National Institute of Environmental Health Sciences-funded HERCULES Center (P30ES019776). The Ministry of Science and Technology (Taiwan; MOST 109–2621-M-002–021). The Environment Research and Technology Development Fund (JPMEERF15S11412) of the Environmental Restoration and Conservation Agency. The São Paulo Research Foundation (FAPESP). The Science and Technology Commission of Shanghai Municipality (grant number 18411951600). The Estonian Ministry of Education and Research (IUT34–17). A fellowship of Fundação para a Ciência e a Tecnlogia (SFRH/BPD/115112/2016)Medical Research Council UK (grant ID MR/R013349/1), the Natural Environment Research Council UK (grant ID NE/R009384/1), and the EU's Horizon 2020 project, Exhaustion (grant ID 820655). The EU's Horizon 2020 project, Exhaustion (grant ID 820655). The Spanish Ministry of Economy, Industry and Competitiveness (grant ID PCIN-2017–046). The MCIN/AEI/10.13039/501100011033 (grant CEX2018-000794-S). The Career Development Fellowship (number APP1163693) and Leader Fellowship (number APP2008813) of the Australian National Health and Medical Research Council. |
| Zio, 2022 | Burkino Faso | Burkina Faso | Temperature, humidity and insolation | COVID-19 | West Africa | Spearman's rank correlation, Multi-output Gaussian process (single and multi-output) | A significant correlation between the daily confirmed COVID-19 cases was found with humidity, wind direction, wind speed, and insolation. These parameters are used to construct the predictive model using the Multi-Output Gaussian process (MOGP). Different combinations of the data of meteorological parameters together with the data of daily reported COVID-19 cases were used to derive different models. We found that the best predictor is obtained using the combination of humidity and insolation. This model was then used to predict the daily confirmed COVID-19 cases knowing the humidity and insolation. | None reported / none to report |

**Supplementary Material A**

**Glossary with Definitions and Citations**

Bayesian model
A Bayesian model is a statistical framework that incorporates prior knowledge, along with observed data, to estimate the probability distribution of unknown parameters. It updates beliefs through Bayes’ theorem as new evidence becomes available (Gelman et al., 2013).

Computational analysis and modelling
Computational analysis and modelling refer to the use of computer-based algorithms, simulations, and statistical techniques to analyze complex data and represent real-world systems for prediction and decision-making (Winsberg, 2019).

Distributed lag nonlinear model (DLNM)
A DLNM is a statistical approach that assesses associations between an exposure and outcome over a lag period, accounting for both nonlinear and delayed effects. It is frequently applied in environmental epidemiology to examine temperature–health relationships (Gasparrini, 2014).

Generalised Additive Model (GAM)
A GAM is a flexible regression technique that allows nonlinear relationships between predictors and outcomes by using smooth functions of covariates, often applied in time-series and environmental health studies (Hastie & Tibshirani, 1990).

Logistic regression
Logistic regression is a statistical method used to model the relationship between one or more predictor variables and a binary outcome, estimating the probability of the outcome occurring (Hosmer et al., 2013).

Multiple binary logistic regression
This extension of logistic regression includes multiple independent variables simultaneously to assess their contribution to predicting a binary outcome (Menard, 2002).

Multi-level modelling
Also known as hierarchical linear modelling, multi-level modelling accounts for nested data structures (e.g., individuals within communities) by analyzing data at different levels simultaneously (Raudenbush & Bryk, 2002).

Multivariate meta-regression model
A multivariate meta-regression model extends traditional meta-regression by simultaneously analyzing multiple correlated outcomes or effect sizes, improving estimation and inference (Jackson et al., 2011).

Poisson Generalized Estimating Equations (GEE) model
A Poisson GEE model is used for analyzing correlated count data, accounting for within-cluster correlation through estimating equations rather than likelihood functions (Liang & Zeger, 1986).

RayMan model
The RayMan model is a microclimate model that estimates mean radiant temperature and other thermal comfort indices in complex environments, widely applied in urban climatology and biometeorology (Matzarakis et al., 2010).

Regression analysis and modelling
Regression analysis is a statistical technique used to examine the relationship between dependent and independent variables, with regression modelling applied to prediction, inference, and hypothesis testing (Montgomery et al., 2021).

Satellite Inversion model
A satellite inversion model retrieves atmospheric or surface properties (e.g., air pollution levels, land surface temperature) from satellite remote-sensing data using physical or statistical inversion algorithms (Li et al., 2017).

Time series analysis
Time series analysis involves statistical methods for analyzing data points collected over time to identify trends, cycles, and autocorrelation, often applied in economics, climatology, and epidemiology (Chatfield, 2003).

Time-to-event analysis
Also known as survival analysis, this method estimates the time until an event occurs, accounting for censored data where the event has not been observed for some subjects (Kleinbaum & Klein, 2012).

Vector Error Correction Model (VECM)
A VECM is an econometric model used to capture both short-term dynamics and long-term equilibrium relationships among non-stationary time-series variables that are cointegrated (Johansen, 1991).

**References**

Chatfield, C. (2003). *The analysis of time series: An introduction* (6th ed.). Chapman & Hall/CRC.

Gasparrini, A. (2014). Modeling exposure–lag–response associations with distributed lag non-linear models. *Statistics in Medicine, 33*(5), 881–899. <https://doi.org/10.1002/sim.5963>

Gelman, A., Carlin, J. B., Stern, H. S., Dunson, D. B., Vehtari, A., & Rubin, D. B. (2013). *Bayesian data analysis* (3rd ed.). CRC Press.

Hastie, T. J., & Tibshirani, R. J. (1990). *Generalized additive models*. Chapman & Hall.

Hosmer, D. W., Lemeshow, S., & Sturdivant, R. X. (2013). *Applied logistic regression* (3rd ed.). Wiley.

Jackson, D., White, I. R., & Riley, R. D. (2011). Quantifying the impact of between-study heterogeneity in multivariate meta-analyses. *Statistics in Medicine, 31*(29), 3805–3820. <https://doi.org/10.1002/sim.5453>

Johansen, S. (1991). Estimation and hypothesis testing of cointegration vectors in Gaussian vector autoregressive models. *Econometrica, 59*(6), 1551–1580. <https://doi.org/10.2307/2938278>

Kleinbaum, D. G., & Klein, M. (2012). *Survival analysis: A self-learning text* (3rd ed.). Springer.

Li, T., Shen, H., Yuan, Q., Zhang, X., & Zhang, L. (2017). Estimating ground-level PM2.5 by fusing satellite and station observations: A geo-intelligent deep learning approach. *Geophysical Research Letters, 44*(23), 11,985–11,993. <https://doi.org/10.1002/2017GL075710>

Liang, K. Y., & Zeger, S. L. (1986). Longitudinal data analysis using generalized linear models. *Biometrika, 73*(1), 13–22. <https://doi.org/10.1093/biomet/73.1.13>

Matzarakis, A., Rutz, F., & Mayer, H. (2010). Modelling radiation fluxes in simple and complex environments: Application of the RayMan model. *International Journal of Biometeorology, 54*(2), 131–139. <https://doi.org/10.1007/s00484-009-0261-0>

Menard, S. (2002). *Applied logistic regression analysis* (2nd ed.). Sage.

Montgomery, D. C., Peck, E. A., & Vining, G. G. (2021). *Introduction to linear regression analysis* (6th ed.). Wiley.

Raudenbush, S. W., & Bryk, A. S. (2002). *Hierarchical linear models: Applications and data analysis methods* (2nd ed.). Sage.

Winsberg, E. (2019). *Philosophy and climate science*. Cambridge University Press.
